# Deletion of the 2-acyl-glycerophosphoethanolamine cycle improve glucose metabolism in *Escherichia coli* strains employed for overproduction of aromatic compounds

**DOI:** 10.1186/s12934-015-0382-6

**Published:** 2015-12-01

**Authors:** César Aguilar, Noemí Flores, Fernando Riveros-McKay, Diana Sahonero-Canavesi, Susy Beatriz Carmona, Otto Geiger, Adelfo Escalante, Francisco Bolívar

**Affiliations:** Departamento de Ingeniería Celular y Biocatálisis, Instituto de Biotecnología, Universidad Nacional Autónoma de México (UNAM), 62210 Cuernavaca, Morelos Mexico; Winter Genomics, Manizales 906, Colonia Lindavista, Delegación Gustavo A. Madero, 07300 México D.F., México; Centro de Ciencias Genómicas, UNAM, Apdo. Postal 565-A, 62210 Cuernavaca, Morelos Mexico

**Keywords:** Metabolic engineering, Metabolic plasticity, PTS system, *Escherichia coli*, Adaptive laboratory evolution, 2-Acyl-glycerophosphoethanolamine cycle, Glycerol metabolism, Glucose metabolism

## Abstract

**Background:**

As a metabolic engineering tool, an adaptive laboratory evolution (ALE) experiment was performed to increase the specific growth rate (µ) in an *Escherichia coli* strain lacking PTS, originally engineered to increase the availability of intracellular phosphoenolpyruvate and redirect to the aromatic biosynthesis pathway. As result, several evolved strains increased their growth fitness on glucose as the only carbon source. Two of these clones isolated at 120 and 200 h during the experiment, increased their μ by 338 and 373 %, respectively, compared to the predecessor PB11 strain. The genome sequence and analysis of the genetic changes of these two strains (PB12 and PB13) allowed for the identification of a novel strategy to enhance carbon utilization to overcome the absence of the major glucose transport system.

**Results:**

Genome sequencing data of evolved strains revealed the deletion of chromosomal region of 10,328 pb and two punctual non-synonymous mutations in the *dhaM* and *glpT* genes, which occurred prior to their divergence during the early stages of the evolutionary process. Deleted genes related to increased fitness in the evolved strains are *rppH*, *aas*, *lplT* and *galR*. Furthermore, the loss of *mutH*, which was also lost during the deletion event, caused a 200-fold increase in the mutation rate.

**Conclusions:**

During the ALE experiment, both PB12 and PB13 strains lost the *galR* and *rppH* genes, allowing the utilization of an alternative glucose transport system and allowed enhanced mRNA half-life of many genes involved in the glycolytic pathway resulting in an increment in the μ of these derivatives. Finally, we demonstrated the deletion of the *aas*-*lplT* operon, which codes for the main components of the phosphatidylethanolamine turnover metabolism increased the further fitness and glucose uptake in these evolved strains by stimulating the phospholipid degradation pathway. This is an alternative mechanism to its regeneration from 2-acyl-glycerophosphoethanolamine, whose utilization improved carbon metabolism likely by the elimination of a futile cycle under certain metabolic conditions. The origin and widespread occurrence of a mutated population during the ALE indicates a strong stress condition present in strains lacking PTS and the plasticity of this bacterium that allows it to overcome hostile conditions.

**Electronic supplementary material:**

The online version of this article (doi:10.1186/s12934-015-0382-6) contains supplementary material, which is available to authorized users.

## Background

Adaptation based on mutations is essential to bacterial survival during environmental challenges, such as exposure to antibiotics, variations in oxygen levels or temperature, and during limited availability of carbon [1–4]. Bacterial adaptation that makes living cells extraordinarily plastic is quick and evident within shorts periods of time [1–3], where often beneficial mutations occur that confer increased fitness in new conditions.

**Fig. 1:**
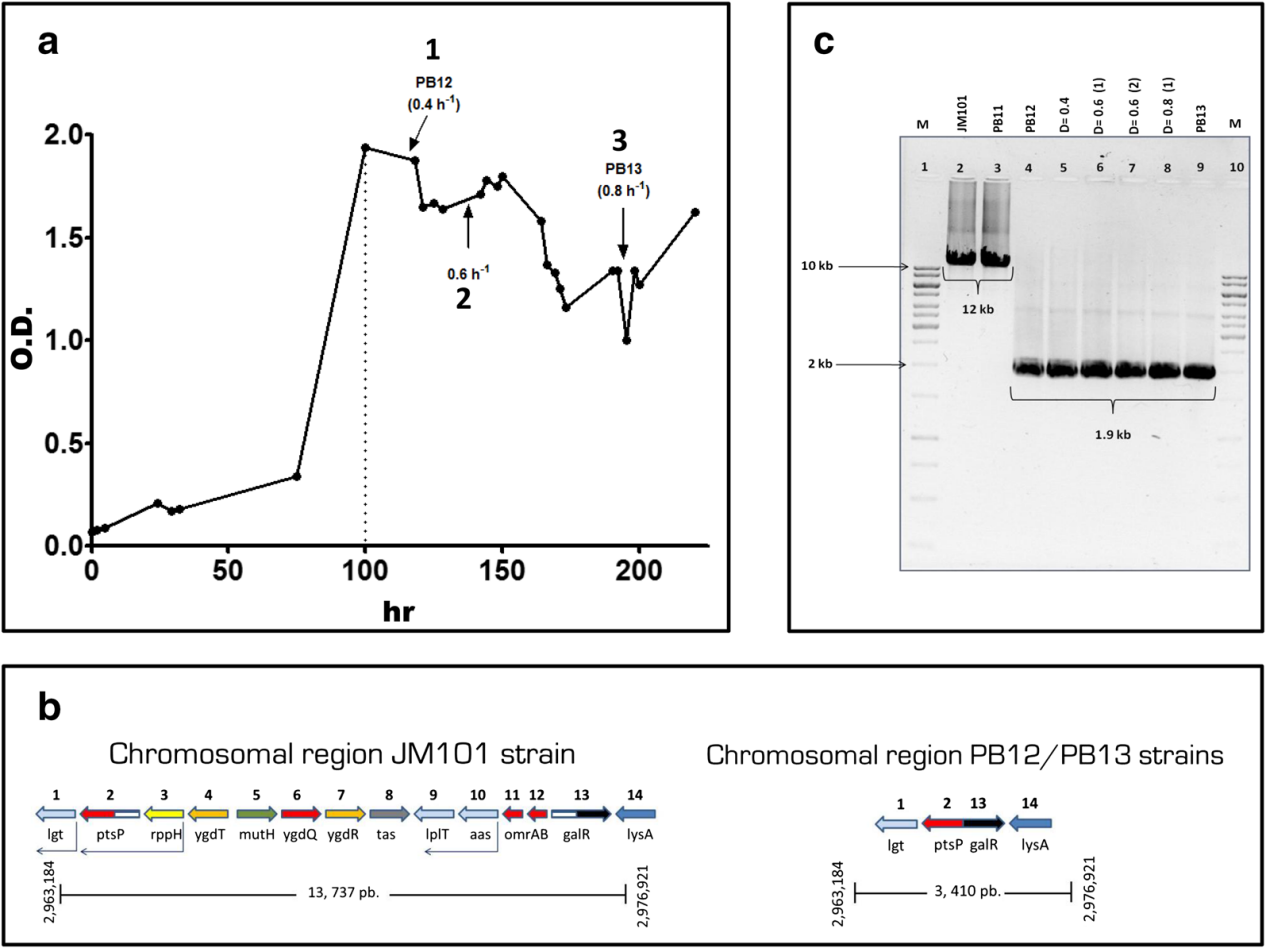
Adaptive laboratory experiment. **a** Isolation of evolved strains from a continuous culture of the PB11 from Aguilar et al. [2]. The *arrows* indicate the isolation time for various strains including PB12 and PB13 strains. *Dotted line* indicates the end of the batch culture and the start of the continuous culture; *numbers* indicate dilution rates (D = h^−1^) as follows: 1 for D = 0.4, 2 for D = 0.6 and 3 for D = 0.8. **b** Chromosomal gene organization in the parental wild type JM101 strain and in the laboratory evolved PB12 and PB13 strains. **c** PCR test for the chromosomal deletion in the evolved strains isolated at D = 0.4, 0.6 and 0.8, where the absence of the 10,328 bp chromosomal DNA fragment can be observed in 6 strains. *Line 1*, (M) molecular weight marker; *lines 2* and *3*, amplification of the chromosomal region in the JM101 and PB11 strains controls respectively (12 kb); *lines 4*–*9*, amplification of the 1.9 kb from the chromosomal region in the six strains isolated during the continuous culture as follows: *line 4* and *5*, PB12 and the second strain isolated at D = 0.4, *line 6* and *7*, first and second strains isolated at D = 0.6, *line 8* and *9*, first strain isolated at D = 0.8 and PB13 strain; *line 10*, (M) molecular weight marker

The adaptive capacity of bacteria can be exploited by metabolic engineering to enhance a particular characteristic in a microorganism [1, 2, 5]. A short-term adaptive laboratory evolution (ALE) process has been performed in our group to increase the diminished growth capacity of an *E. coli* strain (PB11) lacking the major glucose uptake system, phosphoenolpyruvate: carbohydrate phosphotransferase system (PTS). This PTS^−^ strain shows a specific growth rate (μ) of 0.1 h^−1^ and was generated after *ptsHIcrr* operon inactivation in the JM101 wild type parental strain that grows with a μ of 0.7 h^−1^ on glucose as the only carbon source [6–8]. Despite the low growth capacity using glucose as the unique carbon source in the PB11 strain, this strategy diverts a large proportion of the phosphoenolpyruvate (PEP) to the aromatic biosynthesis pathway. However, because of its diminished growth rate, PB11 is not useful as an industrial production strain. Therefore, an adaptive laboratory evolution (ALE) experiment was carried out with this strain by growing in a fermentor with glucose as the only carbon source fed at progressively higher rates (Fig. 1a). During this process, spontaneous mutants that grew faster on glucose were isolated. Further characterization of two strains obtained at 120 h (PB12) and 200 h (PB13) during the fermentation process showed increased μs of 338 and 373 %, respectively, compared to the parental PB11 strain (Fig. 1a) [2, 6–8]. Because of this enhanced capacity, mainly the PB12 strain has been used for the overproduction of aromatic compounds with high yields [9–11].

These two evolved strains have been studied extensively. Among their main characteristics are that glucose uptake is performed by the GalP permease and phosphorylated by the Glk glucokinase. Additionally, they have an increased glycolytic flux compared to the parental PB11 strain and the co-utilization capacity of several carbon sources because of the absence of catabolic repression due to the lack of the PTS system, as previously described [6, 8, 12, 13]. In addition, whole genome sequence analyses of the PB12 strain allowed for the identification of 23 non-synonymous and 22 synonymous point mutations, as well as a 10,328 bp chromosomal deletion. The effects of some of these genetic changes on the growth of the PB12 strain were also reported [2].

**Fig. 2:**
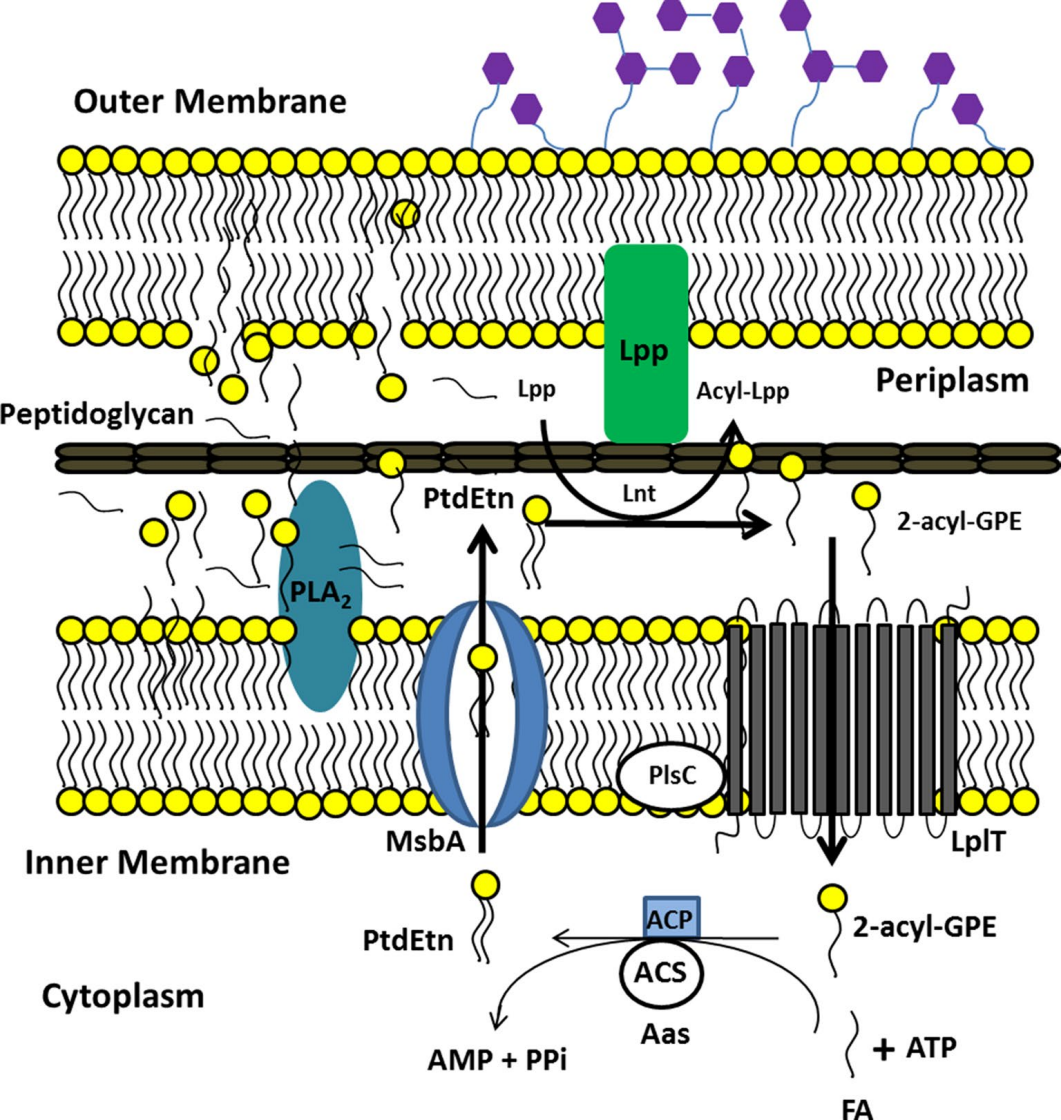
The 2-acyl-GPE cycle. The 2-acyl-GPE cycle initiates at the periplasmic side of the inner membrane with the conversion of PtdEtn into 2-acyl-GPE by the apolipoprotein *N*-acyltransferase Lnt which transfers the fatty acid moiety from the *sn*-1 position of phosphatidylethanolamine (PtdEtn) to the N terminus of the major outer membrane lipoprotein (Lpp). The 2-acyl-GPE is then transported to the cytoplasm through LplT protein. Once inside, the PtdEtn molecule is regenerated by the action of the 2-acyl-GPE acyltransferase/acyl-ACP synthethase Aas protein by the re-acylation of the 2-acyl-GPE molecule. Then the PtdEtn molecule is exported through the MsbA protein to the periplasmic side of the inner membrane. The abbreviations are as follows: phosphatidylethanolamine (PtdEtn), 2-acyl-glycerophosphatidylethanolamine (2-acyl-GPE), fatty acid (FA), Lipoprotein (Lpp), acylated lipoprotein (Acyl-Lpp), Apolipoprotein *N*-acyltransferase (Lnt), phospholipase A type 2 (PLA_2_), lipopolysaccharide transporter (MsbA), lysophospholipid transporter (LplT), 1-acylglycerol-3-phosphate *O*-acyltransferase (PlsC), 2-acylglycerophosphoethanolamine acyltransferase/acyl-ACP synthetase (Aas), acyl-ACP synthetase activity (ACP), 2-acylglycerophosphoethanolamine acyltransferase activity (ACS), Adenosine triphosphate (ATP), Adenosine monophosphate (AMP), inorganic pyrophosphate (PPi)

In the PB12 strain, was also deleted the *aas*-*lplT* operon during the ALE as part of the deletion of 10,328 bp chromosomal region [2]. The products of this operon are involved in the 2-acyl-glycerophosphoethanolamine cycle (2-acyl-GPE). In wild type *E. coli* cells, the 2-acyl-GPE cycle initiates with the transfer of the fatty acid moiety at the 1-position of phosphatidylethanolamine (PtdEtn) to the N-terminus of the major outer membrane lipoprotein (Lpp), resulting in 2-acyl-GPE formation (Fig. 2). Later, the 2-acyl-GPE is transported to the cytosolic side of the cell by the lysophospholipid transporter (LpIT) protein, coded by the *lplT* gene [14]. Once inside the cell, the bifunctional 2-acyl-GPE acyltransferase/acyl-ACP synthetase (Aas), coded by the *aas* gene, re-acylates the 2-acyl-GPE molecule using acyl-ACP as the acyl donor to regenerate PtdEtn. This is then exported by the lipopolysaccharide transporter MsbA (coded by the *msbA* gene) (Fig. 2). Because of the chromosomal deletion in the PB12 strain, this cycle is not functional.

To determine if both the PB12 and PB13 evolved strains followed similar or independent adaptive paths and obtain a deeper insight into the genetic changes that these evolved strains developed to overcome the absence of the major glucose transport system, in this contribution whole genome sequencing of PB13 strain was carried out. Additionally, RT-qPCR measurements of central carbon metabolism, phospholipid degradation genes and the evaluation of growth and metabolite concentration of key mutants were determined. Results indicate that an early loss of the chromosomal region of ~10 kb is also present in the PB13 strain. In addition, two genes with punctual non-synonymous mutations in *dhaM* and *glpT* are present in both PB12 and PB13 strains. It is possible that after the occurrence of these genetic changes, a divergence event took place between the two PTS derivative strains. Derived from these changes we propose a novel metabolic strategy to contend with carbon stress that takes advantage of the degradation PtdEtn pathway in these strains. We also describe and compare the genetic basis of growth adaptation of PB12 and PB13 derivative *E. coli* variants, resulted from the same short term ALE process, that shows differential yields in the production of aromatic compounds, derived from specific genetic changes.

## Results and discussion

### Detection and description of the genetic changes in the PB12 and PB13 strains

Comparative genome analysis of the PB13 strain  carried out by Winter Genomics Inc., shows that similar to the PB12 strain genome, several point mutations that are present in the evolved PB12 and PB13 strains were generated during the ALE process compared to the PB11 parental strain.

In the PB12 strain, 23 non-synonymous and 22 synonymous point mutations were identified [2], while in the PB13 strain, 13 non-synonymous (Table 1) and 7 synonymous point mutations were detected (Additional file 1: Table S1). Additionally, the same 10,328 nucleotide deleted region from the chromosome in the PB12, was detected in the PB13 strain, removing the *rppH*, *ygdT*, *mutH*, *ygdQ*, *ygdR*, *tas*, *lplT*, *aas*, *omrA*, *omrB,* and part of *ptsP* and *galR* genes (Table 1). The analysis of the point mutations in non-coding regions (data not shown) and the synonymous point mutations that were detected in the PB12 strain [2] and in PB13 are not included in this work because the non-coding point mutations are located outside regulatory regions, and the synonymous mutations unlikely have any significant effect on the physiology of the strains (Additional file 1: Table S1).

**Table 1:** Mutations that occurred in the evolved PB13 strain during the ALE process

*Gene*	Basic description	Mutations			
		JM101	PB13	Nucleot.	Pos/Chan
(A) JM101 vs PB13 Non-synonymous point mutations (WG)					
	Hypothetical/putative/unknown function genes				
*wzyE*	Predicted Wzy protein involved in ECA polysaccharide chain elongation	GCG	GtG	221	74 A-V
*ydiM*	Predicted transporter of the major facilitator superfamily (MFS) of transporters	TTC	cTC	982	328 F-L
*ydaF*	Predicted protein	ACT	AaT	116	39 T-N
	Metabolism and transport				
*bglA*	One of several 6-phospho-β-glucosidases in *E. coli*	CCG	CtG	839	280 P-L
*deoB*	Phosphodeoxyribomutase; catabolic enzyme of the pyrimidine deoxyribonucleosides degradation pathway	GAC	GgC	848	283 D-G
*dhaM*	*Dihydroxyacetone kinase subunit M, homologous to certain PTS components*	*TGg*	*TGa*	*1038*	*346 W-stop*
*glpT*	*Major uptake transporter for glycerol-3-phosphate, belongs to the major Facilitator Superfamily (MFS)*	*CCG*	*CtG*	*416*	*139 P-L*
*ispG*	IspG, catalyzes the sixth step in the methylerythritol phosphate pathway	GTC	GcC	749	250 V-A
*mepA*	Penicillin-insensitive d-alanyl-d-alanine (DD) endopeptidase	CGT	CaT	197	66 R-H
*msbA*	ATP-binding lipopolysacchride transport complex	ACG	gCG	367	123 T-A
*xapB*	Xanthosine MFS transporter	ACC	gCC	1051	351 T-A
*ydcT*	YdcT is an ATP-binding component of a predicted spermidine/putrescine ABC transporter	CCG	CtG	113	38 P-L
	Cellular constituents				
*fliN*	FliN, is one of three components of the flagellar motor’s “switch complex”	GAT	GgT	371	124 D-G
	Regulatory genes				
*polA*	DNA Polymerase I (Pol I)	GCG	GtG	2084	695 A-V
(B) JM101 vs PB13 Deletions (WG)					
*ptsP*	Member of a second PTS chain involved in nitrogen metabolism	Pres	Abs	–	–
*rppH*	*RNA pyrophosphohydrolase that initiates mRNA degradation by hydrolysis of the 5′-triphosphate end*	*Pres*	*Abs*	–	–
*ygdT*	Hypothetical protein	Pres	Abs	–	–
*mutH*	*dGATC endonuclease in the MutHLS complex, the methyl-directed mismatch repair pathway*	*Pres*	*Abs*	–	–
*ygdQ*	Putative transport protein	Pres	Abs	–	–
*ygdR*	Predicted protein	Pres	Abs	–	–
*tas*	Putative NAD(P)-linked reductase that acts in starvation-associated mutations	Pres	Abs	–	–
*lplT*	Lysophospholipid transporter (LplT)	Pres	Abs	–	–
*aas*	2-acylglycerophosphoethanolamine acyltransferase/acyl-ACP synthetase	Pres	Abs	–	–
*omrA*	Small RNA that is involved in regulating the protein composition of the outer membrane	Pres	Abs	–	–
*omrB*	Small RNA that is involved in regulating the protein composition of the outer membrane	Pres	Abs	–	–
*galR*	*DNA-binding transcription factor; represses transcription of the operons involved in transport and catabolism of D-galactose*	*Pres*	*Abs*	–	–

A: presents the 13 non-synonymous point mutations in structural genes that changed the code for a different amino acid when compared to the parental PB11 and JM101 strains genomes. In addition 8 synonymous point mutations in different genes were also detected (Additional file 1: Table S1). The non-synonymous point mutations *dhaM* and *glpT* shared by PB12 [4] and PB13 strains are in italic letters. B: presents lost genes in the chromosomal region deleted. Basic descriptions of these genes were taken from http://www.ecocyc.org. *WG* Winter Genomics Inc.

## Non-synonymous point mutations in the evolved strains

From the 13 non-synonymous point mutations detected in the PB13 strain, 2 are located on genes with putative or unknown function, 9 on genes involved in metabolism or transport, and the last 2 mapped in genes involved in motility and its regulation (Table 1). One interesting mutation originated in the *polA* gene, which codes for DNA polymerase I that is involved in several DNA repair pathways [15–17]. This mutation resulted in a valine instead of an alanine codon at position 695 of the protein inside of the Klenow fragment, which is involved in proofreading activity (Table 1). This mutation is located in a non-conserved region [18, 19]; however, this polymerase changes its conformational state according to its different roles, implicating several amino acids [20, 21]. In that sense, it is possible that this modification could have an effect on the polymerase proofreading activity.

Two relevant mutations are located in the *dhaM* and *glpT* genes and were detected in both evolved strains. The *dhaM* gene codes for the DhaM protein, which is a component of the dihydroxyacetone kinase (DhaK). This component consists of three PTS homologous domains (EI, Hpr and the A domain of the EII protein) [22]. The mutation generates an opal stop codon (TGA) instead of a tryptophan codon (TGG) at position 346 of the protein (Table 1), which results in a truncated protein in the EI homologous domain.

**Fig. 3:**
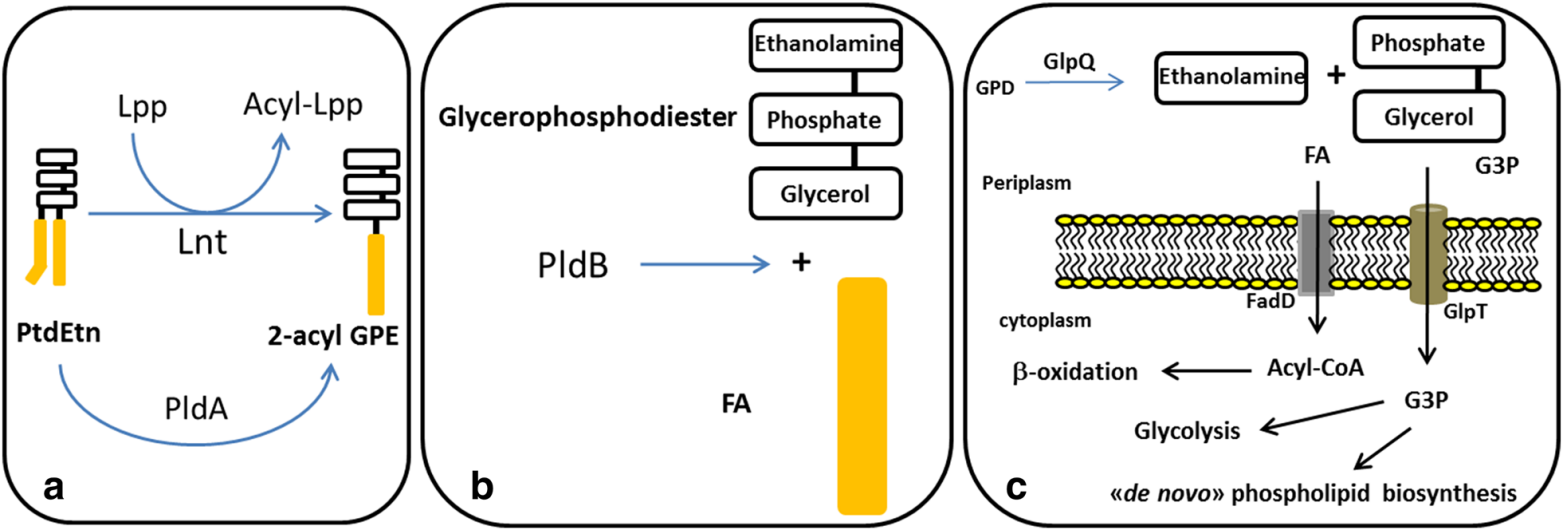
Phosphatidylethanolamine (PtdEtn) degradation pathway in *E. coli*. **a** The degradation pathway initiates with the conversion of PtdEtn into 2-acyl-GPE by the apolipoprotein *N*-acyltransferase Lnt as shown in Fig. 2, or by the outer membrane phospholipase A PldA. **b** After the 2-acyl-GPE formation, in the periplasm, the lysophospholipase L2 PldB, hydrolyzes the bond to the remaining acyl group of the lysophospholipid, generating a fatty acid and glycerophosphodiester molecules. **c** Later, the glycerophosphoryl diester phosphodiesterase GlpQ enzyme, hydrolyzes the glycerophosphodiesters molecules into G3P and ethanolamine. The products of the degradation, G3P and FA are internalized by the cell through the GlpT and FadD transporters and can be further metabolized through the glycolytic and β-oxidation pathway, respectively. *E. coli* can also use ethanolamine as source of carbon and nitrogen [37]; however, a transporter is not described yet. The abbreviations are as follows: phosphatidylethanolamine (PtdEtn), 2-acyl-glycerophosphatidilethanolamine (2-acyl-GPE), fatty acid (FA), Glycerol-3-phosphate (G3P), glycerophosphodiester (GPD), Lipoprotein (Lpp), acylated lipoprotein (Acyl-Lpp), apolipoprotein *N*-acyltransferase (Lnt), phospholipase A type 2 (PLA_2_), lysophospholipase L2 (PldB), glycerophosphoryl diester phosphodiesterase (GlpQ), fatty acyl-CoA synthetase (FadD), glycerol-3-phosphate:phosphate antiporter (GlpT)

The other shared punctual mutation was on the *glpT* gene, which is co-transcribed with the *glpQ* gene. The glycerophosphoryl diester phosphodiesterase (GlpQ) protein encodes a periplasmic glycerophosphoryl diester phosphodiesterase activity (GDP), which hydrolyzes deacylated phospholipids (glycerophosphoalcohol from phospholipids) to an alcohol and glycerol-3-P (G3P) that is subsequently transported into the cell by the glycerol-3-phosphate:phosphate antiporter (GlpT) transporter (Fig. 3) [23]. GlpT is the major G3P uptake system in *E. coli* [24]; this protein consists of 12 transmembrane α-helices, seven cytoplasmic, and six periplasmic regions [25]. The mutation is located in a conserved region on the fourth transmembrane α-helix, generating a leucine instead of a proline codon (Table 1). Interestingly, even though the ALE was carried out on glucose as the only carbon source, two point mutations appeared on genes involved in the glycerol metabolism pathway in both PTS^−^ strains, suggesting a possible role for these two proteins during growth recovery on glucose.

In the PB13 strain, the appearance of a point mutation was noted in the *msbA* gene, which codes for the lipopolysaccharide transporter MsbA. This protein is involved in the translocation of PtdEtn to the periplasmic side of the inner membrane once this molecule has been recycled from 2-acyl-GPE by the Aas protein during the 2-acyl-GPE cycle in the cytoplasm [14] (Fig. 2). The mutation is located at position 123 of the protein in the transmembrane domain [26] and resulted in the substitution of a threonine by an alanine residue. Depletion or loss of function of MsbA results in the accumulation of lipopolysaccharide and phospholipids in the inner membrane of *E. coli* [27]. The relevance of this particular mutation on the physiology of this strain is in principle of low impact in the turnover of PtdEtn because the genes encoding two proteins (Aas and LplT) involved in the 2-acyl-GPE cycle were lost during the ALE process. However, its occurrence, together with the *aas*-*lplT* operon deletion (discussed later) suggests that the adaptation in the PB12 and PB13 strains was directed towards the loss of function of the 2-acyl-GPE cycle.

According with their functions is unlikely that the remaining genes in PB13 with non-synonymous point mutations have an impact on fitness (Table 1).

## Chromosomal deletion during the short term ALE process

As mentioned and reported for the PB12 strain [2], the deletion of a 10,328 bp chromosomal fragment including 12 contiguous genes (*rppH*, *ygdT*, *mutH*, *ygdQ*, *ygdR*, *tas*, *lplT*, *aas*, *omrA*, *omrB,ptsP* and *galR)* was detected in both PB12 and PB13 evolved strains. The deletion limits mapped within the *ptsP* and *galR* genes, resulting in a fusion of the remaining segments of these two genes (Fig. 1b). Earlier work describing the effect on several central metabolic gene transcripts and its impact on the glycolytic pathway allowing for better glucose uptake due to the absence of the *rppH* gene has been published [2]. The detection of the same missing region in the PB13 chromosome establishes the importance of the adaptive nature of this DNA fragment deletion and proves that both strains were derived from the same ancestor.

## Evolutionary divergence between the PB12 and PB13 strains

During the ALE experiment, strain PB11 was grown in a batch culture fermentor containing M9 minimal medium supplemented with 2 g/L of glucose (Fig. 1a). Under these conditions, a selection pressure was generated, favoring mutants that acquired the capacity to grow faster than the original PB11 parental strain. The batch culture was maintained until the stationary phase before starting a continuous culture, in which fresh medium was fed at progressively higher dilution rates (Fig. 1a) [2, 6, 7]. This procedure allowed for the isolation of the mutants generated during the batch culture according to their growth rates. Because two strains were isolated at different dilution rates during the continuous culture (0.4, 0.6 and 0.8 h^−1^), we tested for chromosomal integrity in six strains, including PB12 and PB13. All clone variants lacked the same chromosomal fragment (Fig. 1c), indicating that all strains were derived from the same ancestor. This strongly suggests that the chromosomal deletion is an important early adaptive event in the evolutionary process of this PB11 strain. Interestingly, this same deletion was detected in other evolved strains derived from a duplicate of the ALE experiment using the same PB11 strain (data not shown).

Once the derivatives acquired a growth advantage due to the loss of this specific chromosomal fragment, the cells were able to increase their μ and continued to accumulate mutations with an increased capacity due to the absence of the *mutH* gene that was also lost in the deletion. Although the precise emergence of *dhaM* and *glpT* point mutations is unknown, it is probable that these mutations were generated after the chromosomal deletion event because the cell has to overcome the absence of its principal phospholipid turnover mechanism, and these mutations could have a significant role to contend with this lost capacity, as discussed later (Figs. 2, 3). In addition, we tested for the presence of *dhaM* and *glpT* mutations in all the other intermediate strains and found both in all of them (data not shown). The shared mutations in the PB12 and PB13 strains establish that the divergence between PB12 and PB13 took place after the chromosomal deletion and the appearance of the *dhaM* and *glpT* point mutation events, likely at early stages of the exponential growth phase.

**Fig. 4:**
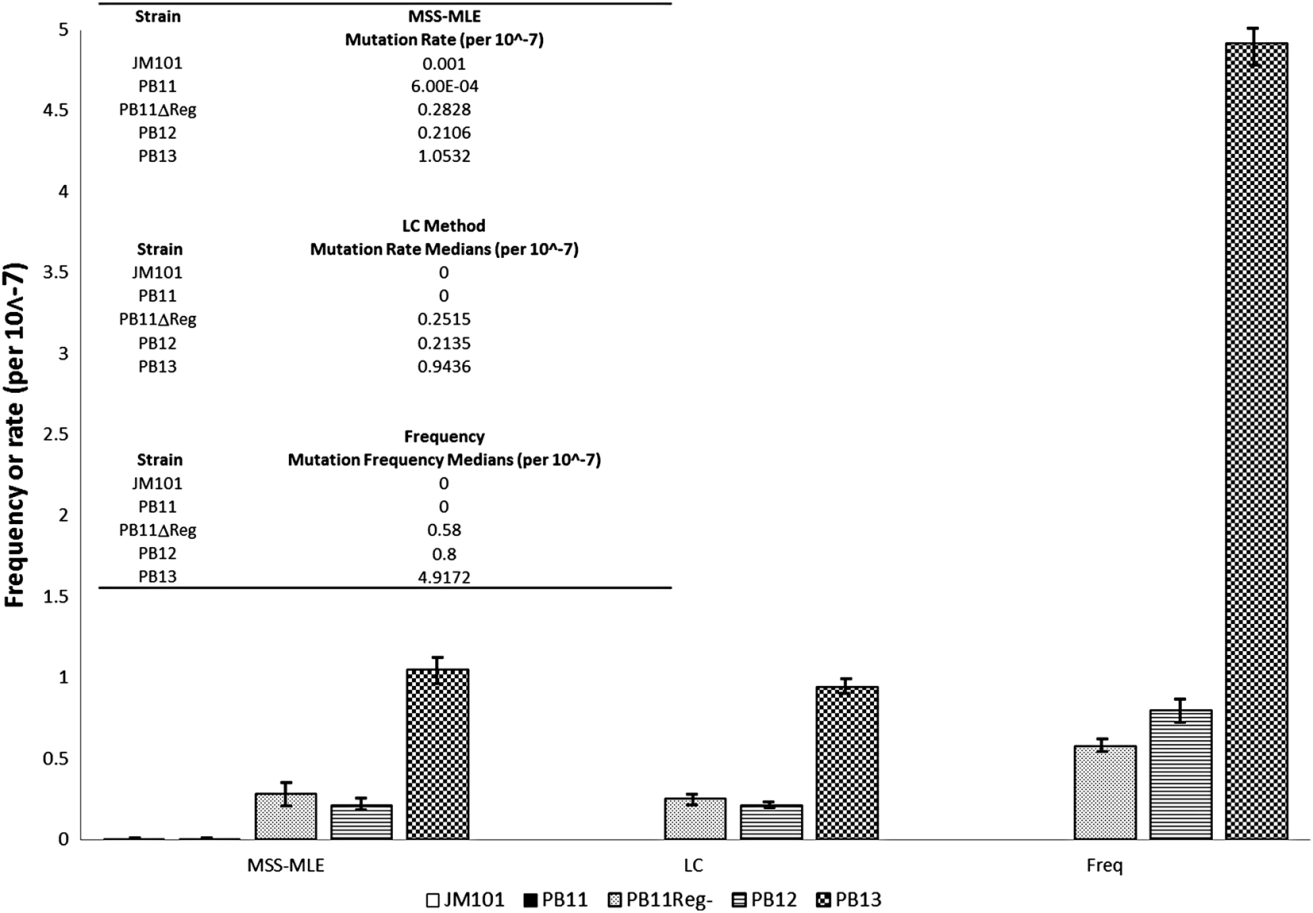
Mutation rates test. Mutation rates in the laboratory evolved strains and its parentals were determined by a modified Luria-Delbrück fluctuation test, employing the MSS-MLE and the LC methods  in the estimation of the number of mutants. The mutation rate is higher in the PB12 and PB13 strains as compared to JM101 and PB11 strains. Mutation frequency was also calculated (Freq). All the results have a confidence interval of 95 %. A detailed table is available in the Additional file 2: Table S2

With the enormous stress that the absence of the major glucose transport system represents, a mutagenic response was triggered in the population, which in turn resulted in better adaptation capacities [1, 2, 28]. Therefore, during this ALE process, the deletion of this important chromosomal fragment was originated in the parental PB11 strain. This event implied the appearance of a population with a higher mutation rate than the wild type due to the absence of the *mutH* gene, which was located in the missing chromosomal region and is involved in DNA repair pathways [29]. The mutation rate in both evolved strains was measured based on the mutation rate in the *rpsL* locus (streptomycin-resistant mutants). Mutation rates were determined by a modified Luria-Delbrück fluctuation test, employing the Ma-Sandri-Sarkar maximum likelihood method (MSS-MLE) and the Lea-Coulson method of the median (LC) in the estimation of the number of mutants [30–32]. As expected, increased mutation rates were observed in the evolved strains due to the deletion of *mutH*. However, a 5-fold higher mutation rate was detected in the PB13 strain compared to the PB12 strain (Fig. 4; Additional file 2: Table S2). A PB11ΔReg derivative (which lacks the entire chromosomal fragment deleted in the evolved strains) showed a similar mutation rate to that of the PB12 strain, so it is feasible to propose that the PB13 strain carries at least one additional mutation that increases its mutation rate. Interestingly, the point mutations detected in the PB12 strain were higher than the observed in the PB13 despite having a lower mutation rate. Since all five previously isolated evolved strains did not carry the *polA* mutation (data not shown), a possible explanation is that the *polA* mutation, has arisen during the last stages of the evolutionary process, and is responsible for the higher mutation rate in the PB13 strain.

## Contribution of the absent chromosomal fragment on growth rate

**Fig. 5:**
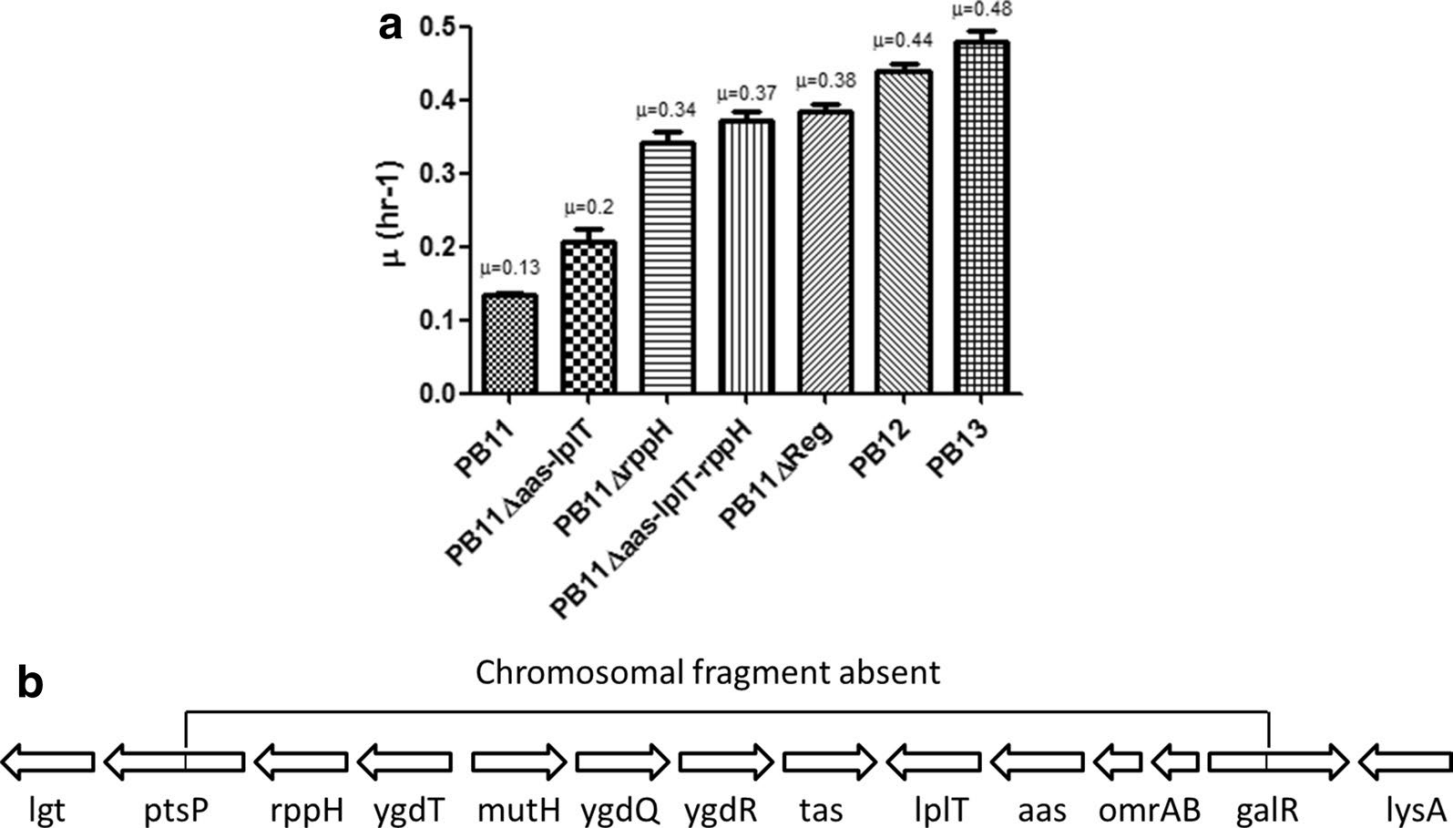
Contribution of the deletion of the chromosomal fragment on growth rate in derivatives of the PB11 strain. **a** The specific growth rates (μ) on glucose as the only carbon source of different PB11 derived strains were determined in order to establish the contribution of some absent genes over the growth in a PTS^−^ strain. **b** The gene order inside the chromosomal fragment is shown

It has been reported that in strain PB12, the absence of the *rppH* and *galR* genes are important for growth rate increases on glucose [2]. To establish the contribution of additional genes within the deleted chromosomal fragment on the growth rate of the evolved strains (Fig. 5b), four different PB11 derived strains were constructed (Fig. 5a). In these derivative strains, *rppH* (as previously reported for PB12 strain [2]), *aas*-*lplT*, or the entire chromosomal fragment were eliminated. The selection of these genes was made after considering their functions and its possible effect on growth (mRNA lifetime for the *rppH* gene as previously reported, as well as PtdEtn turnover for the *aas*-*lplT* operon). A 154 % growth increase was observed in the PB11Δ*aas*-*lplT* strain, while 261 % and 285 % growth increases were observed in the PB11Δ*rppH* and PB11Δ*aas*-*lplT*-*rppH* double mutant derivatives, respectively, where an epistatic effect was evident (Fig. 5a). Finally, the PB11ΔReg had a similar μ compared to the PB11 double mutant (0.38 h^−1^ vs 0.37 h^−1^, respectively), confirming the epistasis observed previously (Fig. 5a). With these results, it is possible to establish that the absence of the *rppH* gene, the *aas*-*lplT* operon, and the *galR* gene are primarily responsible for the increased μ on glucose in the evolved strains. Other important data from these results imply that the point mutations in the PB12 and PB13 strains have only a minor impact on growth recovery (Fig. 5a). Therefore, the growth increase due to the loss of the chromosomal fragment contributes approximately 86 % in PB12 and 79 % in PB13, while the point mutations in PB12 and PB13 contribute only 14 % and 21 %, respectively.

## Importance of the mutated *glpT* and *dhaM* genes in the evolved strains

As was mentioned (Table 1), the two evolved strains share two point mutations in two different genes, suggesting an adaptive role To understand the physiological importance of these genes during the growth on glucose (see Figs. 2, 3), a complete set of single mutants in all strains was constructed. As seen in Table 2, the complete inactivation of the *glpT* gene decreased the μ in both evolved strains. A growth reduction of 43 and 47 % was observed in PB12 and PB13, respectively. Interestingly there was no growth reduction in the PB11 or JM101 strains.

**Table 2:** Specific growth rates of the JM101, PTS^−^ strains and their derivatives with important inactivated genes

Strain	μ (h^−1^)	%
JM101	0.71	100
JM101Δ*glpT*	0.74	104
JM101Δ*dhaM*	0.71	100
JM101Δ*glpQ*	0.72	101
JM101Δ*fadD*		
PB11	0.13	100
PB11Δ*glpT*	0.13	100
PB11Δ*dhaM*	0.11	85
PB11Δ*glpQ*	0.13	100
PB11Δ*fadD*	0.12	92
PB11Δ*aas*-*lplT*-*fadD*	0.12	92
PB12	0.44	100
PB12Δ*glpT*	0.25	57
PB12Δ*dhaM*	0.37	84
PB12Δ*glpQ*	0.26	60
PB12Δ*fadD*	0.32	73
PB13	0.47	100
PB13Δ*glpT*	0.25	53
PB13Δ*dhaM*	0.28	60
PB13Δ*glpQ*	0.26	55
PB13Δ*fadD*	0.29	62

We also evaluated the *glpQ* gene, which is co-transcribed with *glpT* and is involved in the same phospholipid degradation pathway (Figs. 2, 3). Very similar results to those observed with the elimination of *glpT* occurred. Elimination of the *glpQ* gene did not affect the growth of the parental JM101 and PB11 strains, but a 40 and 45 % of growth reduction was observed in the PB12 and PB13 evolved strains, respectively (Table 2). These results strongly suggest the participation of this pathway in the growth recovery of the evolved strains. In agreement with this, both evolved strains lack the *aas*-*lplT* operon that is involved in membrane phospholipid recycling, which supports this last hypothesis.

To evaluate the contribution of the second shared punctual non-synonymous mutation on the physiology of the PTS^−^ strains, individual Δ*dhaM* mutants were generated in all strains (Table 2). The results showed a decrease in the μ of all the PTS^−^ mutant strains, especially in the evolved ones. The strain PB11 had a slight decrease in its μ, which is not considered significant. However, the PB12 and the PB13 strains had decreases of 16 and 40 %, respectively (Table 2). These results suggest that the DhaK could play an important role in the growth of the evolved strains, particularly in the PB13 strain. Interestingly, this protein (similar to the GlpT transporter) is involved in the glycerol pathway, which suggests that the phospholipid-glycerol-3-P degradation pathway contributes to the fitness increase in both evolved strains (Fig. 3). Nevertheless, it is unclear how this mutation provides an advantage because this mutation should decrease the phosphorylation capacities of the protein, which in turn would reduce the conversion of dihydroxyacetone (DHA) to dihydroxyacetone phosphate (DHAP) that serves as an intermediary in the glycolytic pathway. Another important point is that under glycolytic conditions, wild type cells are not able to produce DHA because this compound is generated from glycerol and not from glucose. It is possible that the consumption of G3P from the phospholipid metabolism in the evolved strains could be related to this behavior, but we are unable to completely explain the possible effect on cell physiology.

## Advantage and contribution of the absence of the *aas*-*lplT* operon on the growth rate of the evolved strains

The phospholipids in the membrane of *E. coli* are phosphatidylethanolamine (PtdEtn) (75–80 %), phosphatidylglycerol (PtdGro) (15–20 %), and cardiolipin (CL) (~6 %) [33, 34]. PtdEtn can be metabolized by two pathways. The first route involves the recycling of this molecule by the 2-acyl-GPE cycle, in which all of its principal constituents are involved in a cycle and are not degraded further. The second metabolic pathway involves the complete degradation of the phospholipid molecule to G3P, fatty acids, and ethanolamine (Figs. 2, 3) and that could function in both evolved strains.

This second pathway in wild type cells requires the sequential activity of the apolipoprotein N-acyltransferase Lnt or the phospholipase PldA to generate a 2-acyl-GPE (Fig. 3a) and the inner membrane phospholipase PldB to generate a glycerophosphodiester and free fatty acid molecules from 2-acyl-GPE (Fig. 3b). Later in this pathway, the GDP protein coded by the *glpQ* gene hydrolyzes glycerophosphodiester to generate ethanolamine and G3P (Fig. 3c). The G3P molecule is in turn internalized from the periplasmic space to the cytoplasm through the GlpT transporter that is coded by the *glpT* gene [35]. The free fatty acids formed during PldB action are converted to CoA derivatives by the activity of fatty acyl-CoA synthetase (FadD) [36]. The G3P molecule is then redirected by the cell towards glycolysis or the *de novo* phospholipid biosynthesis pathway. The wild type cell could send fatty acids towards β-oxidation or to the phospholipid synthesis pathway (Fig. 3c). *E. coli* can also use ethanolamine as source of carbon and nitrogen [37]; however, a transporter has not been described.

It was previously reported that *E*. *coli* mutants with completely inactivated *lplT* and *aas* genes have a diminished PtdEtn turnover, resulting in an increase of periplasmic 2-acyl-GPE that is approximately 1/3 higher in the Δ*lplT* mutants compared to the wild type strain. The accumulation of 2-acyl-GPE is higher in the Δ*aas* Δ*pldB* double mutants [14], which suggests a central role for the proteins with lysophospholipase A_2_ activity (PldB) on the alleviation of the 2-acyl-GPE accumulation by the secondary degradation route. It has also been reported that Δ*aas* mutants have an altered membrane phospholipid composition and accumulate both 2-acyl-GPE and acyl-phosphatidylglycerol (acyl-PtdGro) [38]. The acyl-PtdGro molecule is the result of the addition of a fatty acid from 2-acyl-GPE to the molecule of PtdGro by the lysophospholipase A_2_ PldB protein, establishing an additional fate for the 2-acyl-GPE that is modified to glycerol-3-phosphoethanolamine (a substrate for the GDP enzyme) (Figs. 2, 3).

With these elements, it is likely that 2-acyl-GPE and/or acyl-PtdGro derived from PtdEtn could be accumulated in the periplasmic space in the PB12 and PB13 evolved strains compared to the wild type parental JM101 due to the absence of their recycling machinery (Aas and LplT). Therefore, to understand the lipid metabolism in the PB12 and PB13 strains, lipids were analyzed by thin-layer chromatography (TLC). The results showed no significant differences between all the strains (data not shown) suggesting the use of an alternative pathway in the evolved strains, thus preventing the accumulation of lyso-phospholipids species, and an important metabolic plasticity in these strains.

**Fig. 6:**
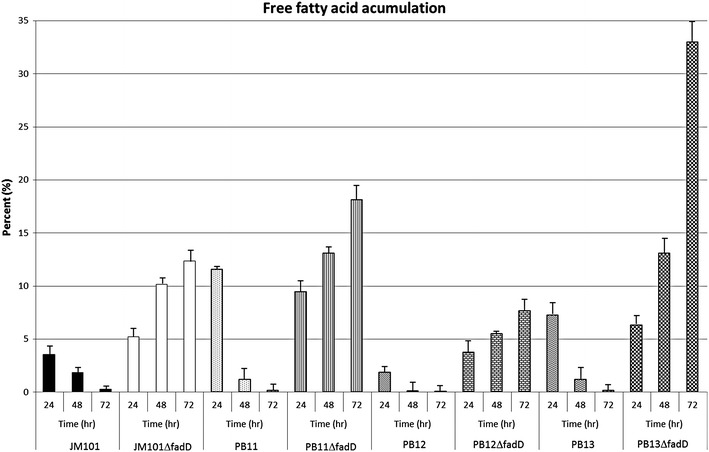
Accumulation of free fatty acids in different *E. coli* strains. *In vivo labeling* of *E. coli* using [^14^C]acetate and after thin-layer chromatographic separation of lipids was performed. The accumulation of free fatty acids of the different Δ*fadD* *E. coli* JM101, PB11, PB12 and PB13 derivative strains at 24, 48 and 72 h of growth were determined. The experiments were made by triplicate. In all strains lacking the *fadD* gene, a progressive increase of free fatty acid was observed over time due to a lack of consumption. The PB11Δ*fadD* and PB13Δ*fadD* strains reached 18 and 33 % of the total lipidic species after 72 h. Interestingly, the PB12Δ*fadD* reached around 8 % of free fatty acids while the JM101Δ*fadD* reached about 12 % in 72 h of growth. The control strains are capable to consume completely the generated free fatty acids during the growth

**Fig. 7:**
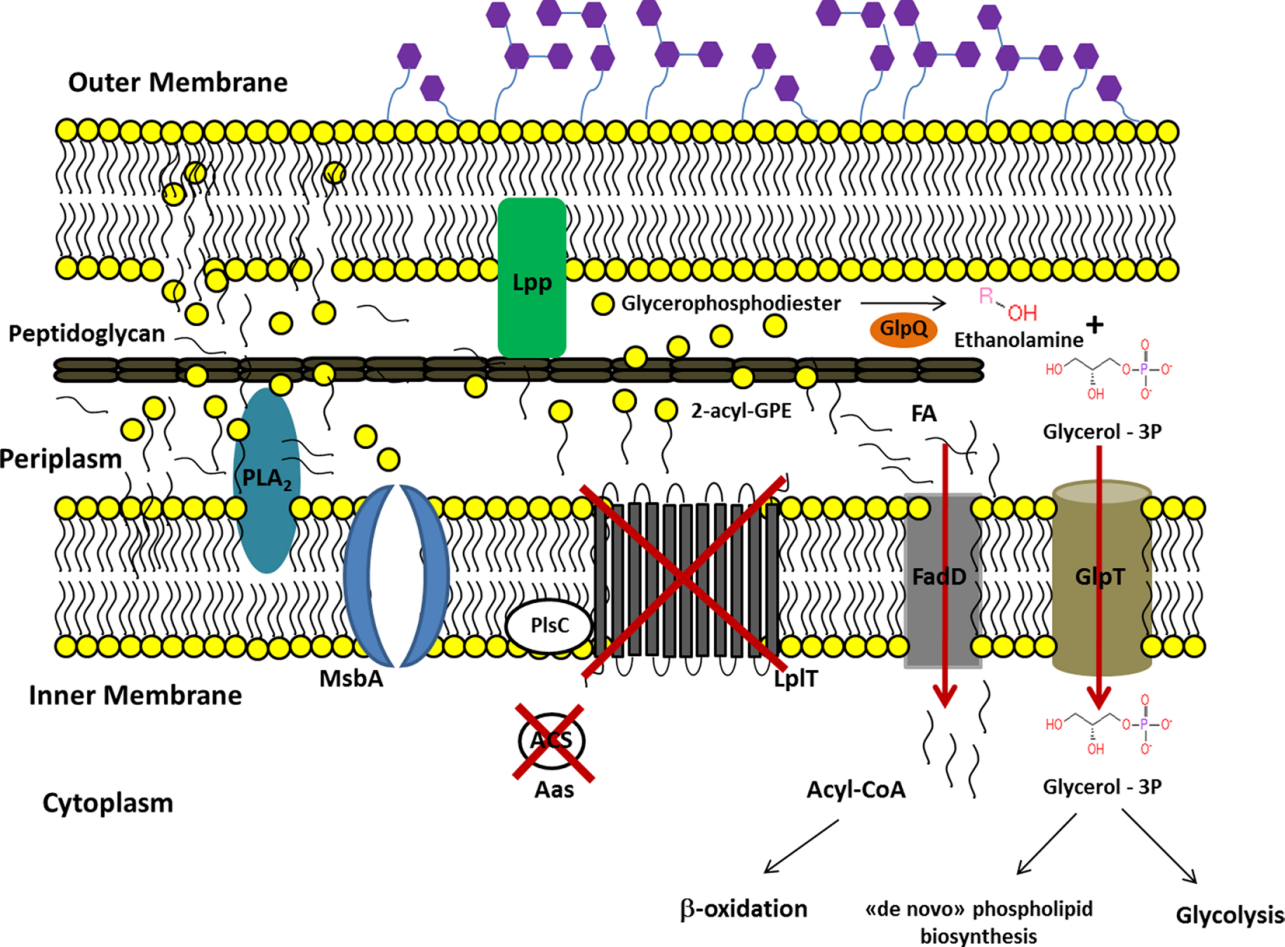
Metabolic strategy in evolved strains lacking the *aas*-*lplT* operon. Proposed phosphatidylethanolamine (PtdEtn) metabolism model in PB12 and PB13 strains where the deletion of *aas*-*lplT* operon is responsible of the lack of the 2-acyl-GPE cycle that in turn was apparently replaced with the utilization of the degradation pathway. In these evolved strains two additional mutations were detected: in the *glpT* and *dhaM* genes. At least the *glpT* mutation could be playing a role in these strains for PtdEtn utilization and faster growth on glucose. The abbreviations are as follows: phosphatidylethanolamine (PtdEtn), 2-acyl-glycerophosphatidylethanolamine (2-acyl-GPE), fatty acid (FA), Lipoprotein (Lpp), acylated lipoprotein (Acyl-Lpp), Apolipoprotein *N*-acyltransferase (Lnt), phospholipase A type 2 (PLA_2_), phosphatidylethanolamine transporter in the 2-acyl-GPE cycle (MsbA), lysophospholipid transporter (LplT), 1-acylglycerol-3-phosphate *O*-acyltransferase (PlsC), 2-acylglycerophosphoethanolamine acyltransferase/acyl-ACP synthetase (Aas), acyl-ACP synthetase activity (ACP), 2-acylglycerophosphoethanolamine acyltransferase activity (ACS), glycerophosphoryl diester phosphodiesterase (GlpQ), fatty acyl-CoA synthetase (FadD), glycerol-3-phosphate:phosphate antiporter (GlpT)

The relative amounts of free fatty acids in *E. coli* reflect a steady state situation defined by formation and consumption of free fatty acids in a given physiological condition. In FadD-deficient mutants, consumption of free fatty acids is eliminated [39] and therefore a more precise estimate of free fatty acid formation can be obtained. To test the utilization of the phospholipid degradation pathway in the PB12 and PB13 evolved strains, the *fadD* gene was inactivated in the different genetic backgrounds. The FadD protein catalyzes the esterification of fatty acids into metabolically active CoA thioesters concomitant with their transport [36], which in this condition are derived from phospholipid degradation (Figs. 6, 7). Inactivation of *fadD* in the PB12 and PB13 evolved strains decreased the μ in these derivatives (Table 2), indicating an important role of this protein in the evolved strains. Therefore, the content of free fatty acids was determined in all the derivative strains. Surprisingly, the JM101Δ*fadD* and the PB11Δ*fadD* strain increased the relative amount of free fatty acids up to 12 and 18 %, respectively after 72 h, suggesting that the degradation pathway is also active in both JM101 and PB11 strains. However, this characteristic does not represent an advantage for the growth of these strains because JM101 is not limited by carbon consumption and in the PB11 the 2-acyl-GPE cycle is present. However, a 33 % increase in the free fatty acid concentration after 72 h in the PB13Δ*fadD* was observed (Fig. 6), confirming the utilization of this degradation pathway in PB13 and suggesting that the lack of the major membrane phospholipid turnover cycle allowed for more efficient carbon metabolism in this strain. The decrease in the growth rate of the Δ*fadD* and the Δ*glpT* mutant strains (Table 2) indicates that this accumulation is due to the PtdEtn degradation pathway. This strategy results in a concomitant optimization of the carbon sources, probably through the elimination of this turnover cycle, which in the PTS^−^ scenario results in a new metabolic capacity to assimilate PtdEtn. This last proposition is supported by the increase in μ in the PB11 strain when the *aas*-*lplT* operon is inactivated (Fig. 5a). In the PB12Δ*fadD* strain, the FA accumulation was approximately 8 % after 72 h (Fig. 6) and the growth decreased by 27 % (Table 2), suggesting that the growth reduction in this strain is due to a different mechanism that could block the pathway rather than a reduction in the re-assimilation products from phospholipid degradation. Therefore, it is feasible that the regulation for this pathway in the PB12 strain works in a slightly different manner. In that sense, non-synonymous point mutations in *yjjU* and *rssA* (which code for predicted esterases) are present in the PB12 strain [2]. Functional and mutational predictions on the proteins coded by these genes in order to evaluate its contribution in the fatty acid accumulation behavior of this strain were carried out (data not shown), however the results are not conclusive.

## RT-qPCR mRNA expression values of central carbon metabolism, PtdEtn degradation, β-oxidation and phospholipid biosynthesis genes in the evolved strains

RT-qPCR analysis of the main central carbon metabolism genes in the PB13 strain was performed. The expression levels of the same genes in the PB11 and PB12 strains were published in previous studies [2, 6, 7]. Interestingly, both evolved strains had similar expression values in most of the genes involved in the metabolic pathways tested (Additional file 3: Figure S1; Additional file 4: Table S3). This indicates that during the early stages of the evolutionary process (minimum 120 h), the main genetic changes responsible for the rearrangement of the expression profile were fixed; among these are the chromosomal fragment deletion and the two non-synonymous point mutations in the *glpT* and *dhaM* genes.

Increased global concentrations of mRNAs were detected due to the absence of *rppH* in PB11Δ*rppH,* PB12 and PB13 strains [2, 6, 7]. However, these higher mRNA concentrations were less intense in both evolved strains (Additional file 3: Figure S1; Additional file 4: Table S3), which suggests that one or more mutations are contributing to these phenomena. Despite the similarities, the higher transcription values of the gene coding for the galactose permease (*galP*) in the PB13 strain should be noted because its value increased 38-fold, while the PB12 increase was approximately 13-fold compare to the parental JM101 strain. Considering the higher growth rate in the PB13 strain (approximately 10 % compared to PB12), it is likely that the increased transcription of the *galP* gene provides an increased glucose uptake in this strain [6].

Supporting the proposition, as result of the activation of the degradation pathway of PtdEtn in the evolved strains, the expression values of the genes involved the degradation of this molecule are increased in the evolved strains, indicating the use of the PtdEtn degradation pathway as a metabolic alternative to contend with the accumulation of lysophospholipid species. On the other hand, most of the values of the PB11 strain were similar to those of the control parental JM101 strain (Table 3). Despite the expected expression profile, there are two interesting aspects to mention. One is regarding the first step in the deacylation of the PtdEtn molecule, which during the 2-acyl-GPE cycle is carried out by the Lnt protein (Fig. 2). The transcripts of the *lnt* gene are increased 2.5-fold only in the PB13 strain. Apparently, during the degradation pathway in the evolved strains, this initial step could be performed mainly by the phospholipase A PldA, coded by the *pldA* gene, which expression values are increased about 3 and 5-fold in the PB12 and PB13 strains, respectively (Table 3). A second remarkable aspect is observed in the expression values of the *glpT* gene, which is the only gene involved in the PtdEtn degradation pathway that increased their mRNA level (2.6-fold) in the PB11 strain. On the other hand, the expression values of this gene for the evolved strains are increased 2.2 and 63.5-fold in the PB12 and PB13 strains, respectively (Table 3), which suggests a high relevance in the reassimilation of G3P for an enhanced metabolism. The unusual overexpression in the PB13 strain for the *glpT* and other genes (as *galP*) reflects the differential regulatory rearrangement in this strain as result of one or the combined effect of more than one genetic change.

**Table 3:** RT-qPCR mRNA expression values of PtdEtn degradation, β-oxidation and phospholipid biosynthesis genes in the evolved strains

Genes	PB11	PB12	PB13
2-Acyl-glycerophosphoethanolamine cycle			
*aas*	1.03 ± 0.05	0	0
*lnt*	1.09 ± 0.26	1.47 ± 0.03	2.43 ± 0.46
*lplT*	0.97 ± 0.08	0	0
*msbA*	1.44 ± 0.08	2.25 ± 0.24	3.87 ± 0.71
PtdEtn degradation			
*fadD*	1.2 ± 0.26	0.66 ± 0.1	0.95 ± 0.26
*glpQ*	1.44 ± 0.39	1.89 ± 0.35	1.90 ± 0.10
*glpT*	2.59 ± 0.40	2.22 ± 0.05	63.46 ± 7.17
*lnt*	1.09 ± 0.26	1.47 ± 0.03	2.43 ± 0.46
*pldA*	1.71 ± 0.30	2.85 ± 0.08	4.79 ± 1.10
*pldB*	0.74 ± 0.05	1.57 ± 0.28	1.65 ± 0.13
β-oxidation			
*fadA*	30.50 ± 0.21	6.83 ± 1.07	7.52 ± 0.71
*fadB*	33.13 ± 2.89	6.20 ± 1.03	4.12 ± 0.69
*fadD*	1.2 ± 0.26	0.66 ± 0.1	0.95 ± 0.26
*fadE*	9.12 ± 1.43	56.93 ± 1.75	13.68 ± 1.56
*fadL*	2.14 ± 0.07	1.34 ± 0.02	2.12 ± 0.53
*fadR*	2.66 ± 0.40	3.00 ± 0.16	4.48 ± 0.22
Fatty acid biosynthesis			
*accA*	1.08 ± 0.20	0.64 ± 0.04	0.62 ± 0.09
*accB*	0.77 ± 0.05	0.78 ± 0.05	0.67 ± 0.10
*accD*	0.64 ± 0.02	0.42 ± 0.04	0.23 ± 0.05
*fabA*	2.25 ± 0.18	3.03 ± 0.16	4.21 ± 0.11
*fabB*	1.99 ± 0.22	1.26 ± 0.12	2.63 ± 0.33
*fabD*	1.25 ± 0.03	1.50 ± 0.05	1.95 ± 0.11
*fabF*	0.66 ± 0.06	1.54 ± 0.05	2.88 ± 0.04
*fabG*	0.69 ± 0.01	1.38 ± 0.11	1.74 ± 0.07
*fabH*	1.02 ± 0.19	1.30 ± 0.24	2.05 ± 0.21
*fabI*	2.04 ± 0.13	5.01 ± 0.20	3.00 ± 0.00
*fabR*	0.90 ± 0.06	1.72 ± 0.11	2.12 ± 0.28
*fabZ*	0.59 ± 0.07	1.84 ± 0.01	1.91 ± 0.24
Phospholipid biosynthesis			
*plsB*	2.30 ± 0.13	2.12 ± 0.50	2.52 ± 0.31
*plsC*	0.52 ± 0.06	1.67 ± 0.12	2.35 ± 0.18
*plsX*	2.39 ± 0.15	2.34 ± 0.28	2.55 ± 0.08

Relative mRNA concentrations of PB11, PB12 and PB13 strains, grown on glucose as the sole carbon source were determined by RT-qPCR. Data in this table are reported as relative expression levels of the parental strain JM101. The mRNA level of each gene in the parental strain was used as control to normalize the data, assigning it the value of one. Central carbon metabolism genes and other important genes have been previously reported and are listed in Additional file 4: Table S3

Regarding the expression of genes involved in β-oxidation, high transcripts values were observed in all the strains, especially for the PB11 strain. This strain presents a permanent stress condition due to its inability for glucose uptake. As a result, carbon scavenging responses are triggered that allow the induction of several catabolic pathways [6–8]. As was presented in this contribution (Fig. 6), the PB11 strain accumulated significant levels of free fatty acids, even higher than those observed for PB12, suggesting that this scavenging strategy favors the free fatty acids reassimilation and further degradation in PB11 strain in order to increases the survival chances. It is important to mention that the inactivation of the *aas*-*lplT* operon in the PB11 strain allows a significant μ increase, which indicates that both, the 2-acyl-GPE cycle and the PtdEtn degradation pathways co-exist in this strain. In consequence, after the operon deletion, the 2-acyl-GPE cycle is no longer functional and all the carbon flow is directed through the degradation pathway to be reused as carbon molecules.

The high expression values observed for most of the genes coding for the free fatty acid degradation pathway in the evolved strains (Table 3), indicates as proposed, the reuse of free fatty acid molecules from the PtdEtn degradation pathway, which enhances their metabolism.

The expression values of the genes involved in the fatty acids biosynthetic pathway were also measured. The results show a diminished expression of most of these genes in the PB11 strain. On the other hand, both evolved strains present, high expression values (Table 3). These data, are in agreement with the proposal, since the absence of the 2-acyl-GPE cycle in the evolved strains, force the cells to replenish the fatty acids needed by restoring through this pathway the phospholipids required for a suitable performance. In this regard, it is possible to suggest that the utilization of both pathways in the PB11 results in a waste of carbon molecules, despite the further restoration of phospholipid molecules. Then, the loss of the 2-acyl-GPE cycle and the other mutations that allowed the degradation of PtdEtn in PB12 and the PB13 strains increased the fitness and the growth rate in these evolved derivatives.

## Metabolic strategy in evolved strains lacking the *ptsHIcrr, aas*-*lplT* and *galR* genes

It is important to emphasize the pleiotropic effect of the absence of phospholipid turnover in the evolved and other strains. This absence is clearly advantageous under the PB11 PTS^−^ scenario, where the strain is unable to use glucose efficiently, and a minimal increase in their ability to optimize carbon metabolism is manifested in its growth (PB11Δ*aas*-*lplT*). In the evolved strains, this effect is not evident because both strains enhance glucose uptake through the effects of the *rppH* and *galR* deletions and are not limited for growth on glucose as the PB11 strain [2]. However, the induction of phospholipid degradation in these strains is apparently required for an increased growth rate, as shown by the inactivation of the *fadD* gene. The decrease in growth rate in the PB11Δ*aas*-*lplT*-*fadD* strain also supports this proposal (Table 2).

Supported on the previous results, we propose the follow strategy carried out by the PB11Δ*aas*-*lplT*, PB12 and PB13 strains to overcome the absence of the *aas*-*lplT* operon. The activity of Lnt, but mainly of PldA and PldB are required to degrade PtdEtn and 2-acyl-GPE into fatty acids and glycerol-3-phosphoethanolamine. This latter compound is accumulated in the periplasmic space due to the lack of the LplT transporter (Fig. 7). In a second step, GDP would be responsible for G3P generation from the glycerophosphodiester molecule. With these reactions, the periplasmic fatty acids and G3P concentrations increase while further transportation by the FadD and GlpT proteins, respectively, is carried out (Fig. 7).

Once inside the cell, G3P is available to be redirected towards the glycolytic pathway or to “*novo*” phospholipid biosynthesis. On the other hand, the free fatty acids are also metabolized by the cell and are used to synthesize phospholipids or are degraded during β-oxidation (Fig. 7). This is possible in these strains because of the absence of catabolite repression due to the lack of the PTS system. Therefore, the evolved strains gain the capacity to recycle all the carbon molecules from phospholipid degradation to *denovo* phospholipid biosynthesis, glycolysis, and β-oxidation. These seem to favor the metabolism of evolved strains, allowing for a growth increase and, consequently, resulting in an increased fitness.

## Essential genetic features for the optimization in the aromatic biosynthesis pathway in the PB12 strain

The *ptsHIcrr* deletion to increase the PEP availability is a widely employed strategy in metabolic engineering. The pleiotropic effect from that deletion has been analyzed from several approaches, in order to find a rational solution to overcome, essentially, the limited growth of the PTS^−^ strains. The use of ALE as a tool in this field has allowed to solving this problem, however the appearance of undesirable genetic changes that could bring another unpredictable detrimental effects is an important issue. The evolved strains generated here and its further analysis allowed identifying the main mutations responsibles for uptake and metabolism of glucose. Interestingly, despite both evolved strains shared this mutations (*rppH*, *galR*, and *aas*-*lplT* deletions), further rational genetic modifications to divert the carbon flux towards the aromatic biosynthesis pathway result in a differential aromatic yields [40, 41]. The remarkable overproductive capacity of the PB12 over the PB13 strain was the result of some metabolic properties derived from specific point mutations in both strains.

The overproductive capacity in the PB12 strain comes from a particular uptake rate capacity (5.5 mmol glc h^−1^ gDW) and glucose catabolism that prevent an overflow through the glycolytic pathway, which in turn reduces the waste carbon in the acetic acid form [8]. In addition, the strategies to redirect the carbon flow to the aromatic biosynthesis pathway through the overexpression of key genes diminish even more the acetic acid production [9, 10]. In the other hand, as result of the high levels of *galP* transcripts, the PB13 shows an increased uptake rate (8.5 mmol glc h^−1^ gDW) that generates the overflow responsible of the acetic acid production in similar concentrations compared to the wild type strain (56 % molar percentage) [8]. This particularity in the PB13 strain represent a disadvantage for the aromatic compounds production, however, the genetic changes associated to the higher *galP* gene transcription is unknown.

Interestingly, some efforts in our laboratory to reproduce this metabolic behavior by reverse metabolic engineering approaches in other *E. coli* strains result in low aromatic compounds yields (data not shown), which indicate the importance of the genetic architecture. It seems the intrinsic genetic characteristics of the JM101 strain is desirable for this purpose, however the same fact imply that some other genetic backgrounds could be a better option for optimize the production of aromatic compounds.

## Conclusions

The deletion of a chromosomal fragment containing important genes, such as *rppH*, *mutH*, *galR* and the *aas*-*lplT* operon, is proposed to be one of the early and most important adaptive events during the reported ALE process [2]. The increase of mRNA levels of glycolytic genes because of the RppH pyrophosphohydrolase activity deficiency and the increased glucose uptake through GalP due to the lack of GalR [2, 6–8], in the PB12 and PB13 PTS^−^ evolved strains allows to enhance their glycolytic metabolism. However, despite this growth advantage, the cells also lost their primary membrane phospholipid turnover pathway coded by the *aas*-*lplT* operon. The evolved strains also share two additional point mutations in the *glpT* and *dhaM* genes.

As the inactivations in the *glpT* and *fadD* genes suggests, the lack of the PtdEtn turnover metabolism capacity in the evolved strains was replaced with the utilization of the degradation pathway where phospholipids are transformed to their basic components: G3P and free fatty acids. The increase of more than 50 % in the μ of the derivative PB11Δ*aas*-*lplT* strain supports the adaptive nature of this metabolic pathway blockage. In this situation, the evolved strains additionally improved carbon metabolism by forcing the cell to synthesize fatty acids *de novo* and taking advantage of the energy generated by β-oxidation and glycolysis during the degradation of fatty acids and G3P, respectively. Ultimately, the balance between *de novo* synthesis, β-oxidation, and glycolysis seems to be favorable in both PTS^−^ PB12 and PB13 evolved strains, allowing for an increase in their growth rates on glucose as the only carbon source. These results indicate an important metabolic plasticity of these strains that lack PTS to utilize additional mutations (in *glpT* and *dhaM* genes) that occurred in the ALE to transform the deletion disadvantage of the *aas*-*lplT* operon lost in an advantage for growing faster on glucose by utilizing the capacity of recycling phospholipids as additional carbon and energy resources (Fig. 7). The RT-qPCR values of the genes involved in the capacity of recycling phospholipids that in general are overexpressed in the PB12 and PB13 evolved strains, are in agreement with this proposed recycling capacity that is enhanced and functional in the evolved strains and mainly in PB13 (Table 3).

The high increase in the growth rate observed with the deletion of *rppH*, *aas*, *lplT* and the entire chromosomal region in PB11 derivatives indicates that the contribution to the growth recovery by point mutations is minimal compared to the benefits provided by the chromosomal fragment deletion. However, there is a growth rate difference between both evolved strains caused by the higher *galP* gene expression in the PB13 strain (38-fold increase). It is possible that one of the point mutations present in the PB13 strain could have an indirect role in the increased *galP* transcription observed in this strain. In future experiments, we will individually inactivate these genes to see their effect on *galP* expression and growth rate.

The enormous stress caused by the elimination of PTS triggered a chromosomal rearrangement where the DNA mismatch repair system was affected due to the lack of *mutH* in the deleted DNA fragment. Consequently, the mutation rate increased. The appearance of a mutator population in bacterial cultures is a frequent event during limited growth conditions to improve its chances of survival by generating variation. In these PTS^−^ evolved strains, this genetic novelty displaces alternative subpopulations because it creates in the resulting variants an improved capacity to metabolize glucose due to the absence of the *rppH*, *aas*, *lplT* and *galR* genes, ultimately increasing the fitness of the evolved strains. Interestingly, the absence of this specific chromosomal fragment could have given the cell the capacity to generate variation to explore several adaptive solutions that could overcome the lack of PTS.

Several ALE experiments have demonstrated the efficiency and how fast the evolution works in *E. coli* [2, 5, 42, 43]. This intrinsic feature has been exploited in biotechnology, resulting in a common practice during metabolic engineering strategies to improve particular features in the cell for enhance several bioprocess. In the PB12 and PB13, it was demonstrated that a particular qsGlc derived from specific genetic changes is the decisive factor to determine the efficiency in the aromatic compounds yields. Additionally, some results of preliminary reverse-metabolic engineering approaches to increase the glucose uptake in overproductive aromatic compounds PTS^−^ strains, shows that the genetic architecture of the cell is essential. This issue it should be taking into account in order to enhance the productive capacities of the different metabolites in the industry.

The appearance of a mutator genotype in this kind of metabolic engineering strategies (ALE), allows the cell population to explore different genetic solutions. However, this genetic change in conjunction with the other deleted genes (*galR*, *rppH*, *aas* and *lplT*) resulted in the solution for an increased growth rate. At the moment after the deletion occurred, the cell culture could explore ways to diminish its mutation rate to decrease the probability of gaining deleterious mutations [44, 45], a fact that was not detected in both isolated evolved strains. Rather than decreasing, the PB13 strain increased its mutation rate. Among the genetic changes that could be involved in this increase is the *polA* mutation, which has a role in processing Okazaki DNA fragments and in filling gaps during the excision-repair processes. It is possible that this mutation could affect the performance of the DNA repair process. In the PB12 strain, where the mutation rate is moderate, the chance of generating deleterious mutations is still present. For that reason, it is desirable for stability and production purposes to consider the return of the wild type *mutH* gene, particularly in those PB12 derivative strains that are used for aromatic compound production. Nevertheless, cells where deleterious mutation could have occurred were not detected because of their inability to reproduce. In fact, increasing mutation rates in strains lacking *mutH* allows the cells in this culture to more quickly test several genetic changes (120 h in the reported study) and their effect on survival in different derivative cells.

In fact, an alternative for metabolite production purposes is that the presence of a mutator gene could allow mutations that enhance the production of the specific product. We cannot rule out this possibility in some of the PB12 derivatives that have increased capacities for producing aromatic compounds. For this reason, the genome of some of these strains will be resequenced.

## Materials and methods

### Bacterial strains, growth conditions, and recombinant DNA techniques

*E. coli* strains JM101 [F’ *traD36proAB*^+^*lac*I^q^*lac*ZΔM15/*supE thi* Δ(*lac*-*proAB*) *rpoS(33am)*], PB11 [JM101Δ(*ptsH, ptsI, crr)*:: *kan*], PB12 (PB11ev1, PTS^−^ Glc^+^) and PB13 (PB11ev2, PTS^−^ Glc^+^) derivatives have previously been described [2, 6–8]. The derivatives of these strains utilized in this report (*rppH*, *aas*, *lplT*, *glpT*, *dhaM*, *glpQ* and *fadD* genes were knockout inactivated) were obtained by the Datsenko and Wanner method [46] using the oligonucleotides listed in Table S4 that is available in the Additional file 3: Figure S1. All gene disruptions were confirmed by PCR (data not shown). For *inocula* preparations, strains stored at −72 °C in glycerol were inoculated into Luria broth (LB) for overnight growth.

The cultures of the PB12 and PB13 strains that were utilized for DNA preparation for genome sequencing were obtained from the original culture that was kept frozen in glycerol [2, 7]. For μ determinations, cells were grown in LB and inoculated into M9 minimal medium with 2 g/L of glucose as the only carbon source. When the cultures were growing exponentially, they were inoculated into the same prewarmed (50 mL) medium, using 250 mL baffled flasks at 37 °C and stirred at 300 rpm with a starting optical density at 600 _nm_ (O.D._600nm_) = 0.1. O.D._600nm_ values were measured using a Klett/Summerson photocolorimeter, model 800-3. All specific μ values presented in Table 2 and Fig. 5a are the averages of at least three independent cultures, each one in duplicate. For RNA isolation and RT-qPCR analyses, duplicate cultures were grown in 1 L fermentors using M9 medium with 2 g/L of glucose as the sole carbon source. Cultures were grown at 37 °C, stirred at 600 rpm, and with an air flow rate of 1 vvm with a starting O.D._600nm_ = 0.1. For RT-qPCR determinations, cells from the different fermentations were collected in the log phase at O.D._600nm_ = 1 [6].

### DNA extraction from parental and evolved PB13 strains for genomic analysis

Two overnight cultures of the *E. coli* strains JM101, PB11, and PB13 were grown from their frozen original stocks in liquid LB medium. DNA was extracted with a maxiprep phenol extraction and ethanol precipitation method [2, 6, 47] and purified with the Pure Link PCR purification kit (Invitrogen, USA). Quality and quantity of extracted DNA was verified as recommended by Roche NimbleGen (RN) and by UNAM Massive DNA Sequencing Unit. All strains were sequenced with Illumina Inc. Gallx high throughput technology. The JM101, PB11 and PB13 strains were resequenced with Ion Torrent PGM technology. The nucleotide sequence of the PB12 strain has been previously reported [2].

### Library constructions and sequencing

DNA samples from the JM101, PB11 and PB13 strains were submitted to the Massive DNA Sequencing Unit of UNAM for its paired-ended (PE) library construction and genome sequencing. PE library was constructed following Illumina Inc. recommendations, which were previously described for the nucleotide sequence determination of strain PB12 [2]. The Ion Xpress™ Plus Fragment Library Kit was used for the library construction, and genome sequencing by Ion Torrent PGM was performed following recommendations from Life Technologies.

### Genome *de novo* assembly and variant identification

The protocol used for the “*de novo*” assembly and variant identification from the data obtained by high throughput sequencing with the Illumina GAIIx method was previously described [2]. Ion Torrent reads were trimmed based on their quality using the FASTX-Toolkit (Hannon Lab. FASTX-Toolkit.). These reads were mapped against the JM101 assembly using bwa 0.6.2 [51]. Variants were called using Samtools 0.1.18 [48] and compared with variant calls from the PB13 assembly. Only variants reported by both methods (*de novo* assembly and Ion Torrent read mapping) were reported. For most of the analyses, the Instituto de Biotecnología, UNAM cluster resources were used.

### DNA sequencing of putative mutations by Sanger methodology

DNA regions containing putative mutations detected in the PB12 strain were previously reported [2]. For the PB13 strain, the point mutations detected by variant identification were confirmed. PCR amplifications were performed using the oligonucleotide primers listed in Additional file 5: Table S4 (shown in Additional file 3: Figure S1), purified by the Pure Link PCR purification kit, and sequenced by the Sanger methodology with the Taq FS Dye Terminator Cycle Sequencing Fluorescence-Based Sequencing in a Perkin Elmer/Applied Biosystems Model 3730. Sequence differences in 14 of the mutations are shown in PB13 in Table 1a and were confirmed by examination of the trace data (data not shown).

### Estimation of the mutation rate in the different *E. coli* strains

Determination of mutation rates was based on mutation at a single locus, *rpsL* (rifampicin-resistant mutants). Mutation rates were determined by a modified Luria-Delbrück fluctuation test [30, 31]. Briefly, 100 µL of 30 overnight cultures were independently mixed and plated in LB agar plates containing the antibiotic selector (200 µg/mL of streptomycin). Samples of 100 µL from a 10^−6^ dilution from five overnight cultures were used to estimate the total viable count in drug-free LB plates. The MSS-LME and the LC methods [32] were used to estimate the number of mutants.

### RNA extraction, cDNA synthesis, and RT-qPCR analysis

As previously described, total RNA was isolated and purified using a modified hot phenol method reported elsewhere [2, 6, 7], and a RevertAid™ H minus First Strand cDNA Synthesis kit was used to synthesize cDNA according to the manufacturer’s instructions (Fermentas, Burlington, Canada). For each reaction, approximately 5 μg of RNA and a mixture of 10 pmol/mL of specific DNA reverse primers (b) for each measured gene were used. The nucleotide sequences of these genes in PB12 have been previously published [2, 6, 7] RT-qPCR was performed with the ABI Prism 7300 Real-Time PCR System (Perkin Elmer/Applied Biosystems, Foster City, CA) using the MaximaR SYBR Green/ROX qPCR Master Mix (2x) kit (Fermentas LifeSciences), and reaction conditions were previously described [2]. For each gene, all experiments were performed in triplicate from two different fermentations to obtain very similar values (differences <0.3 SD). A non-template control reaction mixture was included for each gene. Standard curves were constructed to evaluate PCR efficiency, and all the qPCR assays showed a high efficiency of amplification (90–100 %) because the genes had R2 values above 0.9976 with slopes between −3.4 and −3.7. All RT-qPCR experiments were compliant with the MIQE (Minimum Information for Publication of Quantitative Real-Time PCR Experiments) guidelines [49]. The quantification technique used to analyze the data was the 2^−ΔΔCq^ method described by Livak and Schmittgen [50]. Data were normalized using the *ihfB* gene as an internal control.

### *In vivo* labeling of *E. coli* with [^14^C]acetate and analysis of lipid extracts

The lipid composition of the different *E. coli* strains was determined by triplicate following labeling with [1-^14C^]acetate (Perkin Elmer). LB cultures (1.5 mL) were inoculated to an initial optical density at 600 nm (OD_600_) of 0.1 from precultures grown in the same medium. After the addition of 1 μCi/mL [1-^14C^]acetate to each culture, they were incubated for 24, 48 or 72 h. The cells were harvested by centrifugation and resuspended in 100 μL of water. The lipids were extracted according to the method of Bligh and Dyer [51]. The chloroform phase was used for lipid analysis by one-dimensional thin-layer chromatography (TLC) using high-performance TLC silica gel 60 plates (Merck) and ethyl acetate-hexane-acetic acid (60:40:5 [vol/vol/vol]) as the mobile phase. Two-dimensional TLC was performed as described previously [52]. Radioactivity was detected using a Storm 820 PhosphorImager (Amersham Biosciences). Image analysis and signal quantification were carried out using ImageQuant TL (Amersham Biosciences).

## Additional files

10.1186/s12934-015-0382-6 This table lists the data provided by Winter Genomics Inc. (WG) obtained from the whole genome sequence of the PB13 strain in comparison to the parental strains JM101 and PB11. Section A includes a list of 13 genes in which, accordingly to this company non-synonymous point mutations occurred changing the coding regions in structural genes. The table also includes (Section B) the list of 8 genes in which synonymous mutations also occurred.
10.1186/s12934-015-0382-6 Mutation rates in both evolved strains based on mutation appearance in the *rpoB* locus. Mutation rates were determined by a modified Luria-Delbrück fluctuation test, employing the MSS-MLE and the LC methods in the estimation of the number of mutants [30–32]. The mutation frequency also was determinated.
10.1186/s12934-015-0382-6 RT-qPCR values of central metabolism and regulatory genes. Relative mRNA concentrations of central metabolism and regulatory genes of PB12 and PB13 strains, grown in glucose as the sole carbon source were determined by RT-qPCR. The PB12 values have been previously reported [2, 6, 7] and are presented here for discussion and comparison purposes. Data in this Figure are reported as relative expression levels of the parental strain JM101. The mRNA level of each gene in the parental strain was used as control to normalize the data, assigning it the value of one (see Materials and Methods).
10.1186/s12934-015-0382-6 RT-qPCR mRNA expression values of central carbon metabolism, in the evolved strains. Relative mRNA concentrations of central metabolism and regulatory genes of PB11, PB12 and PB13 strains, grown in glucose as the sole carbon source were determined by RT-qPCR. Most of these values have been previously reported (*) and are presented here for discussion and comparison purposes [2, 9–11]. Data in this table are reported as relative expression levels of the parental strain JM101. The mRNA level of each gene in the parental strain was used as control to normalize the data, assigning it the value of one. N.D. Not determined.
10.1186/s12934-015-0382-6 Oligonucleotides employed in this study. This table lists the oligonucleotides utilized in this work. Section A shows the oligonucleotides used for DNA sequencing with the Sanger method, for the gene disruption confirmations generated by Datsenko-Wanner method and the deletion confirmation that occurred in the evolved strains. Section B lists the oligonucleotides utilized for gene disruption with the Datsenko-Wanner methodology. Section C lists the oligonucleotides utilized for RT-qPCR analysis not previously reported.

## References

[1] Lee D-H, Palsson BØ (2010). Adaptive evolution of *Escherichia coli* K-12 MG1655 during growth on a nonnative carbon source, l-1,2-propanediol. Appl Environ Microbiol.

[2] Aguilar C, Escalante A, Flores N, de Anda R, Riveros-McKay F, Gosset G, Morett E, Bolívar F (2012). Genetic changes during a laboratory adaptive evolution process that allowed fast growth in glucose to an *Escherichia coli* strain lacking the major glucose transport system. BMC Genom.

[3] Jang S-H, Kim J, Kim J, Hong S, Lee C (2012). Genome sequence of cold-adapted *Pseudomonas mandelii* strain JR-1. J Bacteriol.

[4] Blázquez J, Couce A, Rodríguez-Beltrán J, Rodríguez-Rojas A (2012). Antimicrobials as promoters of genetic variation. Curr Opin Microbiol.

[5] Minty JJ, Lesnefsky AA, Lin F, Chen Y, Zaroff TA, Veloso AB, Xie B, McConnell CA, Ward RJ, Schwartz DR (2011). Evolution combined with genomic study elucidates genetic bases of isobutanol tolerance in *Escherichia coli*. Microb Cell Fact.

[6] Flores N, Flores S, Escalante A, de Anda R, Leal L, Malpica R, Georgellis D, Gosset G, Bolívar F (2005). Adaptation for fast growth on glucose by differential expression of central carbon metabolism and gal regulon genes in an *Escherichia coli* strain lacking the phosphoenolpyruvate:carbohydrate phosphotransferase system. Metab Eng.

[7] Flores N, Leal L, Sigala JC, de Anda R, Escalante A, Martínez A, Ramírez OT, Gosset G, Bolivar F (2007). Growth recovery on glucose under aerobic conditions of an *Escherichia coli* strain carrying a phosphoenolpyruvate:carbohydrate phosphotransferase system deletion by inactivating *arcA* and overexpressing the genes coding for glucokinase and galactose permease. J Mol Microbiol Biotechnol.

[8] Flores S, Gosset G, Flores N, de Graaf AA, Bolívar F (2002). Analysis of carbon metabolism in *Escherichia coli* strains with an inactive phosphotransferase system by ^13^C labeling and NMR spectroscopy. Metab Eng.

[9] Escalante A, Calderón R, Valdivia A, de Anda R, Hernández G, Ramírez OT, Gosset G, Bolívar F (2010). Metabolic engineering for the production of shikimic acid in an evolved *Escherichia coli* strain lacking the phosphoenolpyruvate: carbohydrate phosphotransferase system. Microb Cell Factories.

[10] Rodriguez A, Martínez JA, Báez-Viveros JL, Flores N, Hernández-Chávez G, Ramírez OT, Gosset G, Bolivar F (2013). Constitutive expression of selected genes from the pentose phosphate and aromatic pathways increases the shikimic acid yield in high-glucose batch cultures of an *Escherichia coli* strain lacking PTS and *pykF*. Microb Cell Factories.

[11] Rodriguez A, Martinez JA, Flores N, Escalante A, Gosset G, Bolivar F (2014). Engineering *Escherichia coli* to overproduce aromatic amino acids and derived compounds. Microb Cell Factories.

[12] Sigala JC, Flores S, Flores N, Aguilar C, de Anda R, Gosset G, Bolivar F (2009). Acetate metabolism in *Escherichia coli* strains lacking phosphoenolpyruvate: carbohydrate phosphotransferase system; evidence of carbon recycling strategies and futile cycles. J Mol Microbiol Biotechnol.

[13] Flores N, de Anda R, Flores S, Escalante A, Hernández G, Martínez A, Ramírez OT, Gosset G, Bolívar F (2004). Role of pyruvate oxidase in *Escherichia coli* strains lacking the phosphoenolpyruvate:carbohydrate phosphotransferase system. J Mol Microbiol Biotechnol.

[14] Harvat EM, Zhang Y-M, Tran CV, Zhang Z, Frank MW, Rock CO, Saier MH (2005). Lysophospholipid flipping across the *Escherichia coli* inner membrane catalyzed by a transporter (LplT) belonging to the major facilitator superfamily. J Biol Chem.

[15] Curti E, McDonald JP, Mead S, Woodgate R (2009). DNA polymerase switching: effects on spontaneous mutagenesis in *Escherichia coli*. Mol Microbiol.

[16] Makiela-Dzbenska K, Jaszczur M, Banach-Orlowska M, Jonczyk P, Schaaper RM, Fijalkowska IJ (2009). Role of *Escherichia coli* DNA polymerase I in chromosomal DNA replication fidelity. Mol Microbiol.

[17] Hastings PJ, Hersh MN, Thornton PC, Fonville NC, Slack A, Frisch RL, Ray MP, Harris RS, Leal SM, Rosenberg SM (2010). Competition of *Escherichia coli* DNA polymerases I, II and III with DNA Pol IV in stressed cells. PLoS ONE.

[18] Dzidic S, Radman M (1989). Genetic requirements for hyper-recombination by very short patch mismatch repair: involvement of *Escherichia coli* DNA polymerase I. Mol Gen Genet MGG.

[19] Orren DK, Selby CP, Hearst JE, Sancar A (1992). Post-incision steps of nucleotide excision repair in *Escherichia coli*. Disassembly of the UvrBC-DNA complex by helicase II and DNA polymerase I. J Biol Chem.

[20] Beese LS, Friedman JM, Steitz TA (1993). Crystal structures of the Klenow fragment of DNA polymerase I complexed with deoxynucleoside triphosphate and pyrophosphate. Biochemistry (Mosc).

[21] Dzantiev L, Romano LJ (2000). A conformational change in *E. coli* DNA polymerase I (Klenow fragment) is induced in the presence of a dNTP complementary to the template base in the active site. Biochemistry (Mosc).

[22] Gutknecht R, Beutler R, Garcia-Alles LF, Baumann U, Erni B (2001). The dihydroxyacetone kinase of *Escherichia coli* utilizes a phosphoprotein instead of ATP as phosphoryl donor. EMBO J.

[23] Larson TJ, Ehrmann M, Boos W (1983). Periplasmic glycerophosphodiester phosphodiesterase of *Escherichia coli*, a new enzyme of the glp regulon. J Biol Chem.

[24] Marger MD, Saier MH (1993). A major superfamily of transmembrane facilitators that catalyse uniport, symport and antiport. Trends Biochem Sci.

[25] Lemieux MJ, Song J, Kim MJ, Huang Y, Villa A, Auer M, Li X-D, Wang D-N (2003). Three-dimensional crystallization of the *Escherichia coli* glycerol-3-phosphate transporter: a member of the major facilitator superfamily. Protein Sci Publ Protein Soc.

[26] Dawson RJP, Locher KP (2006). Structure of a bacterial multidrug ABC transporter. Nature.

[27] Doerrler WT, Raetz CRH (2002). ATPase activity of the MsbA lipid flippase of *Escherichia coli*. J Biol Chem.

[28] Foster PL (2007). Stress-induced mutagenesis in bacteria. Crit Rev Biochem Mol Biol.

[29] Junop MS, Yang W, Funchain P, Clendenin W, Miller JH (2003). *In vitro* and in vivo studies of MutS, MutL and MutH mutants: correlation of mismatch repair and DNA recombination. DNA Repair.

[30] Ma WT, Sandri GV, Sarkar S (1992). Analysis of the Luria-Delbrück distribution using discrete convolution powers. J Appl Probab.

[31] Rosche WA, Foster PL (2000). Determining mutation rates in bacterial populations. Methods San Diego Calif.

[32] Hall BM, Ma C-X, Liang P, Singh KK (2009). Fluctuation analysis CalculatOR: a web tool for the determination of mutation rate using Luria-Delbruck fluctuation analysis. Bioinforma Oxf Engl.

[33] Rilfors L, Lindblom G, Wieslander Å, Christiansson A. Lipid bilayer stability in biological membranes. In: Kates M, Manson LA (eds) Membrane fluidit*y*. Springer, USA. 1984:205–245 **[*****Biomembranes*****, vol. 12]**.

[34] Morein S, Andersson A, Rilfors L, Lindblom G (1996). Wild-type *Escherichia coli* cells regulate the membrane lipid composition in a “window” between gel and non-lamellar structures. J Biol Chem.

[35] Larson TJ, van Loo-Bhattacharya AT (1988). Purification and characterization of *glpQ*-encoded glycerophosphodiester phosphodiesterase from *Escherichia coli* K-12. Arch Biochem Biophys.

[36] Weimar JD, DiRusso CC, Delio R, Black PN (2002). Functional role of fatty acyl-coenzyme A synthetase in the transmembrane movement and activation of exogenous long-chain fatty acids. Amino acid residues within the ATP/AMP signature motif of *Escherichia coli* FadD are required for enzyme activity and fatty acid transport. J Biol Chem.

[37] Garsin DA (2010). Ethanolamine utilization in bacterial pathogens: roles and regulation. Nat Rev Microbiol.

[38] Hsu L, Jackowski S, Rock CO (1991). Isolation and characterization of *Escherichia coli* K-12 mutants lacking both 2-acyl-glycerophosphoethanolamine acyltransferase and acyl-acyl carrier protein synthetase activity. J Biol Chem.

[39] Pech-Canul Á, Nogales J, Miranda-Molina A, Álvarez L, Geiger O, Soto MJ, López-Lara IM (2011). FadD is required for utilization of endogenous fatty acids released from membrane lipids. J Bacteriol.

[40] Báez-Viveros JL, Osuna J, Hernández-Chávez G, Soberón X, Bolívar F, Gosset G (2004). Metabolic engineering and protein directed evolution increase the yield of l-phenylalanine synthesized from glucose in *Escherichia coli*. Biotechnol Bioeng.

[41] Báez-Viveros J, Flores N, Juárez K, Castillo-España P, Bolivar F, Gosset G (2007). Metabolic transcription analysis of engineered *Escherichia coli* strains that overproduce l-phenylalanine. Microb Cell Factories.

[42] Horinouchi T, Suzuki S, Hirasawa T, Ono N, Yomo T, Shimizu H, Furusawa C (2015). Phenotypic convergence in bacterial adaptive evolution to ethanol stress. BMC Evol Biol.

[43] Dragosits M, Mattanovich D (2013). Adaptive laboratory evolution—principles and applications for biotechnology. Microb Cell Factories.

[44] Turrientes M-C, Baquero F, Levin BR, Martínez J-L, Ripoll A, González-Alba J-M, Tobes R, Manrique M, Baquero M-R, Rodríguez-Domínguez M-J, Cantón R, Galán J-C (2013). Normal mutation rate variants arise in a Mutator (Mut S) *Escherichia coli* population. PLoS ONE.

[45] Galán J-C, Turrientes M-C, Baquero M-R, Rodríguez-Alcayna M, Martínez-Amado J, Martínez J-L, Baquero F (2007). Mutation rate is reduced by increased dosage of *mutL* gene in *Escherichia coli* K-12. FEMS Microbiol Lett.

[46] Datsenko KA, Wanner BL (2000). One-step inactivation of chromosomal genes in *Escherichia coli* K-12 using PCR products. Proc Natl Acad Sci U S A.

[47] Ausubel FR (1999). Short protocols in molecular biology.

[48] Li H, Durbin R (2009). Fast and accurate short read alignment with Burrows-Wheeler transform. Bioinforma Oxf Engl.

[49] Bustin SA, Benes V, Garson JA, Hellemans J, Huggett J, Kubista M, Mueller R, Nolan T, Pfaffl MW, Shipley GL, Vandesompele J, Wittwer CT (2009). The MIQE guidelines: minimum information for publication of quantitative real-time PCR experiments. Clin Chem.

[50] Livak KJ, Schmittgen TD (2001). Analysis of relative gene expression data using real-time quantitative PCR and the 2(-Delta Delta C(T)) Method. Methods San Diego Calif.

[51] Bligh EG, Dyer WJ (1959). A rapid method of total lipid extraction and purification. Can J Biochem Physiol.

[52] de Rudder KE, Thomas-Oates JE, Geiger O (1997). *Rhizobium meliloti* mutants deficient in phospholipid N-methyltransferase still contain phosphatidylcholine. J Bacteriol.

