# Supplementary material for: Deletion of the 2-acyl-glycerophosphoethanolamine cycle improve glucose metabolism in Escherichia coli strains employed for overproduction of aromatic compounds
Source: Microb Cell Fact. 2015 Dec 1;14:194. doi: 10.1186/s12934-015-0382-6 (PMC4666226; doi:10.1186/s12934-015-0382-6)
Supplement: Supplementary file 3 — 10.1186/s12934-015-0382-6 RT-qPCR values of central metabolism and regulatory genes. Relative mRNA concentrations of central metabolism and regulatory genes of PB12 and PB13 strains, grown in glucose as the sole carbon source were determined by RT-qPCR. The PB12 values have been previously reported [2, 6, 7] and are presented here for discussion and comparison purposes. Data in this Figure are reported as relative expression levels of the parental strain JM101. The mRNA level of each gene in the parental strain was used as control to normalize the data, assigning it the value of one (see Materials and Methods). [file 12934_2015_382_MOESM3_ESM.pdf]

(PB12) (PB13)

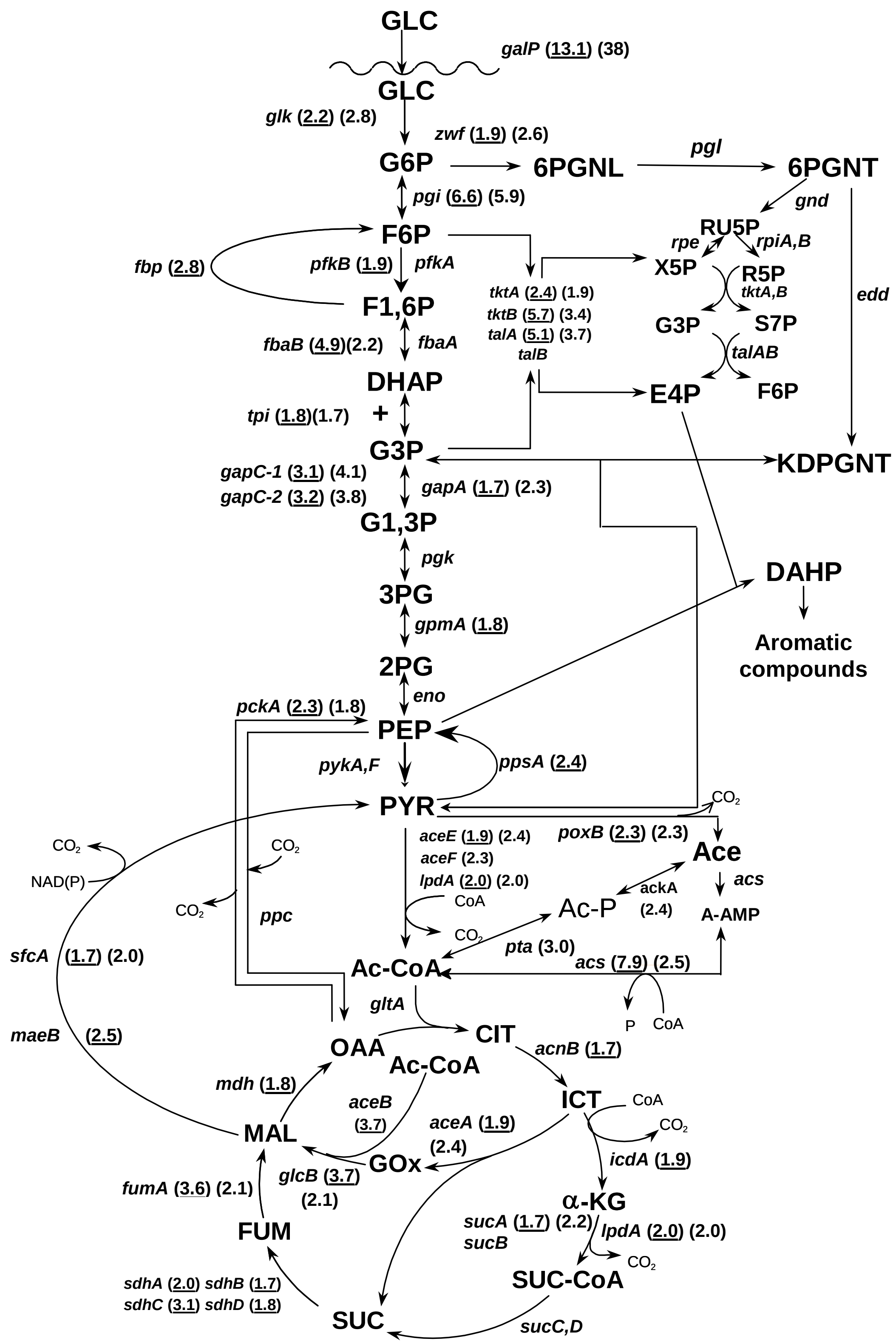

GLC
galP (13.1) (38)
GLC
glk (2.2) (2.8)
zwf (1.9) (2.6)
G6P
6PGNL
pgl
6PGNT
pgi (6.6) (5.9)
gnd
F6P
RU5P
rpe
rpiA,B
X5P
R5P
fbp (2.8)
pfkB (1.9)
pfkA
tktA,B
edd
F1,6P
tktA (2.4) (1.9)
tktB (5.7) (3.4)
talA (5.1) (3.7)
talB
G3P
S7P
fbaB (4.9)(2.2)
fbaA
talAB
DHAP
E4P
F6P
+
tpi (1.8)(1.7)
G3P
KDPGNT
gapC-1 (3.1) (4.1)
gapC-2 (3.2) (3.8)
gapA (1.7) (2.3)
G1,3P
pgk
DAHP
3PG
gpmA (1.8)
Aromatic compounds
2PG
eno
pckA (2.3) (1.8)
PEP
pykA,F
ppsA (2.4)
PYR
CO2
poxB (2.3) (2.3)
Ace
aceE (1.9) (2.4)
aceF (2.3)
lpdA (2.0) (2.0)
acs
CO2
NAD(P)
CO2
CO2
ackA (2.4)
Ac-P
A-AMP
CoA
ppc
CO2
pta (3.0)
acs (7.9) (2.5)
sfcA (1.7) (2.0)
Ac-CoA
gltA
P
CoA
CIT
maeB (2.5)
OAA
acnB (1.7)
Ac-CoA
mdh (1.8)
ICT
CoA
aceB (3.7)
aceA (1.9) (2.4)
CO2
MAL
GOx
icdA (1.9)
glcB (3.7) (2.1)
fumA (3.6) (2.1)
α-KG
sucA (1.7) (2.2)
sucB
lpdA (2.0) (2.0)
FUM
CO2
SUC-CoA
sdhA (2.0) sdhB (1.7)
sdhC (3.1) sdhD (1.8)
SUC
sucC,D

Figure S1. Central metabolic routes showing key metabolites and the genes involved in their transformation. RT-qPCR values of those upregulated genes (1.7-fold or higher) are shown close to the gene underlined in parenthesis for PB12 and in parenthesis for PB13; see Table S3 in additional file 4 for the complete set of RT-qPCR values. The abbreviations are as follows: glucose (GLC), glucose-6-phosphate (G6P), fructose-6-phosphate (F6P), fructose-1,6-phosphate (F1,6P), dihydroxy-acetone phosphate (DHAP), glyceraldehyde-3-phosphate (G3P), glyceraldehyde-1,3-phosphate (G1,3P), 3-phosphoglycerate (3PG), 2-phosphoglycerate (2PG), phosphoenolpyruvate (PEP), pyruvate (PYR), acetyl-CoA (AcCoA), acetyl phosphate (Ac-P), acetyl-AMP (A-AMP), citrate (CIT), isocitrate (ICT), glyoxylate (Gox), a-ketoglutarate (a-KG), succinyl-coenzyme A (SUC-CoA), succinate (SUC), fumarate (FUM), malate (MAL), oxaloacetate (OAA), 6-phosphogluconolactone (6PGNL), 6-phosphogluconate (6PGNT), ribulose-5-phosphate (RU5P), ribose-5-phosphate (R5P), xylulose-5-phosphate (X5P), seudoheptulose-7-phosphate (S7P), erythrose-4-phosphate (E4P), 2-keto-3-deoxy-6-phosphogluconate (KDPGNT), 3-deoxy-D-arabino-heptulosonate-7-phosphate (DAHP).
